# Do tradeoffs among dimensions of women’s empowerment and nutrition outcomes exist? Evidence from six countries in Africa and Asia

**DOI:** 10.1016/j.foodpol.2020.102001

**Published:** 2021-04

**Authors:** Agnes R. Quisumbing, Kathryn Sproule, Elena M. Martinez, Hazel Malapit

**Affiliations:** a International Food Policy Research Institute, Washington, DC, USA; b Sproule Research Group, CA, USA; c Friedman School of Nutrition Science and Policy at Tufts University, Boston, MA, USA

**Keywords:** Women’s empowerment, Gender, Nutrition, Agriculture

## Abstract

Although women’s empowerment and gender equality are often linked with better maternal and child nutrition outcomes, recent systematic reviews find inconclusive evidence. This paper applies a comparable methodology to data on the Women’s Empowerment in Agriculture Index (WEAI), an internationally-validated measure based on interviews of women and men within the same household, from six countries in Africa and Asia to identify which dimensions of women’s empowerment are related to household-, woman-, and child-level dietary and nutrition outcomes. We examine relationships between women’s empowerment and household-level dietary diversity; women’s dietary diversity and BMI; and child-related outcomes, controlling for woman, child, and household characteristics. We also test for differential associations of women’s empowerment with nutrition outcomes for boys and girls. We find few significant associations between the aggregate empowerment scores and nutritional outcomes. The women’s empowerment score is positively associated only with child HAZ, while lower intrahousehold inequality is associated with a higher likelihood of exclusive breastfeeding and higher HAZ but with lower BMI. However, analysis of the subdomain indicators finds more significant associations, suggesting that tradeoffs exist among different dimensions of empowerment. Women’s empowerment accounts for a small share of the variance in nutritional outcomes, with household wealth and country-level factors accounting for the largest share of the variation in household and women’s dietary diversity. In contrast, most of the variation in child outcomes comes from child age. Improving nutritional outcomes requires addressing the underlying determinants of poor nutrition in addition to empowering women and improving gender equality.

## Highlights

• We examine linkages between women’s empowerment and nutrition in 6 countries.
• Women’s empowerment and gender equality are positively associated with child HAZ.
• Tradeoffs exist between domains of empowerment and women’s nutritional outcomes.
• Higher workload is associated with more diverse child diets but lower women’s BMI.
• Women’s empowerment accounts for a small share of the variance in nutrition outcomes.

## 1 Introduction

Women’s empowerment and gender equality are intrinsically important; they also promote household food security and better women’s and children’s nutrition outcomes. Although a large body of empirical evidence documents the links between greater control of resources by women and improved child human capital outcomes (see reviews of observational studies (Quisumbing, 2003) and experimental studies (Yoong, Rabinovich, & Diepeveen, 2012)), a recent systematic review of the role of women’s empowerment in child nutrition outcomes (Santoso et al., 2019), building on Carlson et al., 2015, Cunningham et al., 2015, and Pratley (2016) finds that the relationship between women’s empowerment and child nutrition is inconclusive. Santoso et al. (2019) conclude that the limited evidence is likely not due to the absence of an underlying relationship between women’s empowerment and child nutrition, but to flawed study design. These flaws involve the measurement and aggregation of quantitative indicators of women’s empowerment, complexity in measuring a multidimensional concept, the situational, context-dependent nature of the empowerment process (Pratley, 2016); measurement of autonomy (one’s own influence over choices that affect oneself) and the limitations of cross-cultural comparability (Carlson et al., 2015); and inadequate attention to time allocation, reproductive decisions, and indicators of men’s engagement in women’s empowerment and child nutrition (Santoso et al., 2019).

Conceptual and measurement issues have also stymied the testing of linkages between women’s empowerment and her own nutritional outcomes. Conceptually, these linkages are complex and directions of impact between empowerment and nutrition outcomes are ambiguous. Empowerment itself is affected by other factors, including a woman’s age, education, status within the household and society, and knowledge, all of which directly or indirectly influence nutrition outcomes. Tradeoffs may also exist among various dimensions of empowerment. For example, women may derive increased autonomy from working outside the home, but this may increase her workload and reduce the time available for care responsibilities and for self-care. Kabeer (1999), quoted in Alkire et al. (2013), argues further that there is a gap between the understanding of empowerment as a process and more instrumentalist forms of advocacy that have required the measurement and quantification of empowerment. Quantitative measurements are often done at particular points in time, missing the dynamic nature of the process. Perceptions of empowerment are also context-specific, so women's empowerment indicators have to be sensitive both to the ways that context shapes processes of empowerment, and to whether women are empowered in the specific roles that they play in these particular contexts (Kabeer 1999).

Measurement difficulties have also hindered efforts to assess the strength of the relationship between women’s empowerment and nutrition along the agriculture-nutrition pathways (Ruel & Alderman, 2013). Three of the six agriculture-nutrition pathways identified in a comprehensive review of nutrition-sensitive interventions focus specifically on women: *women’s social status and empowerment* through increased access to and control over resources; *women’s time* through participation in agriculture, which can be either positive or negative for their own and their children’s nutrition; and *women’s health and nutrition* through engagement in agriculture, which may have either positive or negative impacts, depending on exposure to occupational hazards and the balance between energy intake and expenditure (Ruel & Alderman 2013). Although the conceptual framework is widely accepted, empirical evidence on the strength of these relationships, particularly in the context of agriculture-nutrition interventions, remains scarce.

The lack of cross-nationally comparable empowerment measures compounds the measurement difficulties. The most widely available datasets, the Demographic and Health Surveys, focus on household decision-making and decisions in the reproductive, not the productive sphere (Alkire et al., 2013). The availability of a standardized measure of women’s empowerment, the Women’s Empowerment in Agriculture Index (WEAI), which has been collected in large-scale population-based surveys in 19 countries, provides an opportunity to examine how women’s empowerment in the productive sphere in agricultural settings affects food and nutrition security in many contexts. Although relationships between the WEAI and nutrition outcomes have been analyzed in countries as diverse as Bangladesh (Malapit et al., 2019, Sraboni et al., 2014, Sraboni and Quisumbing, 2018), Ghana (Malapit and Quisumbing 2015), India (Gupta, Pingali, & Pinstrup-Andersen, 2019), Kenya (Kassie, Fisher, Muricho, and Diiro 2020), and Nepal (Malapit, Kadiyala, Quisumbing, Cunningham, & Tyagi, 2015), these are single-country studies; relationships between women’s empowerment and nutrition may differ across country contexts, making external validity a concern^1^.

This paper analyzes WEAI data from six countries in Africa and Asia—Bangladesh, Cambodia, Nepal, Ghana, Mozambique, and Tanzania—to identify which indicators and dimensions of women’s empowerment are related to dietary and nutrition outcomes of women, children, and their households and to examine whether tradeoffs exist among these dimensions of empowerment. To our knowledge, this is the first time that data on the WEAI and nutrition outcomes will be analyzed for multiple countries and regions using a comparable methodology. We pool the observations across countries to examine the relationship between women’s empowerment and household dietary diversity, women’s dietary diversity and BMI; and child-related outcomes (exclusive breastfeeding, dietary diversity, and anthropometry), controlling for woman, child, and household characteristics and country fixed effects. We also test whether women’s empowerment has differential associations for boys and girls. We apply a decomposition analysis to examine the contribution of women’s empowerment to nutritional outcomes relative to other covariates, such as individual, household, and country-level characteristics. Finally, we discuss the possible implications for gender-and nutrition-sensitive agricultural programs.

## 2 Empowerment and nutrition: concepts and measures

### 2.1 Empowerment domains and nutrition linkages

Although linkages between increasing resources controlled by women and nutrition are well established (see Quisumbing, 2003, Yoong et al., 2012 for reviews), quantifying the linkages between women’s *empowerment* and nutrition has been more difficult. Each person has a unique definition of empowerment, based on his or her life experiences, personality, and aspirations. Context and culture also shape one’s definition of empowerment. Unsurprisingly, many definitions of empowerment exist (Ibrahim & Alkire, 2007). Kabeer’s (1999) definition, on which the WEAI is based, views empowerment as expanding people’s ability to make strategic life choices, particularly in contexts in which this ability had been denied to them. In this definition, the ability to exercise choice encompasses three dimensions: resources (including not only access but also future claims to material, human, and social resources), agency (including processes of decision-making, negotiation, and even deception and manipulation), and achievements (well-being outcomes) (Kabeer, 1999). The WEAI focuses on agency, which is far less studied than resources (such as income) or achievements (such as educational levels), because it directly addresses the issue of choice or decision-making.

The WEAI also differs from measures of empowerment derived from nationally representative surveys such as some Demographic and Health Surveys, which are based on questions about household decision-making that typically focus on the domestic sphere and do not encompass decisions in the productive and economic spheres. Neither do most surveys have identical questions for men and women (Alkire et al., 2013). Lastly, the WEAI captures control over resources or agency within the agricultural sector, an important source of livelihoods in developing countries that is not covered by most existing indices.

The core concept of the WEAI involves measuring women’s empowerment in five domains in agriculture; assessing men’s empowerment in the same domains gives a measure of the gender gap in empowerment. In this section, we discuss how these domains are operationalized and how they may affect food security and nutrition outcomes.

### 2.2 Domains and indicators of empowerment in the WEAI

WEAI is an aggregate index reported at the country-, region-, or program-level and is composed of two sub-indices: the five domains of empowerment (5DE) and the gender parity index (GPI). The 5DE assesses women’s empowerment in five domains, which include (i) agricultural **production** decisions, (ii) access to and decision-making power over productive **resources**, (iii) control over use of **income**, (iv) **leadership** roles within the community, and (v) **time** allocation. The 5DE is constructed from individual-level empowerment scores reflecting each person’s achievements in the five domains as measured by 10 indicators with their corresponding weights (Table 1). Each binary indicator measures whether an individual has surpassed a given threshold, or has adequate achievement, with respect to each indicator. A woman is defined as empowered if she has adequate achievements in four out of the five domains or has achieved adequacy in 80 percent or more of the weighted indicators.

**Table 1:** Domains, indicators, and weights in the Women’s Empowerment in Agriculture Index.

**Domain**	**Indicator**	**Definition of Indicator**	**Weight**
1. Production	1.1 Input in productive decisions	Sole or joint decision-making over food and cash-crop farming, livestock, and fisheries	1/10
	1.2 Autonomy in production	The extent to which the respondent’s motivation for decision-making about agricultural production reflects own values rather than a desire to please others or avoid harm	1/10
2. Resources	2.1 Ownership of assets	Sole or joint ownership of major household assets	1/15
	2.2 Purchase, sale, or transfer of assets	Whether respondent participates in decision to buy, sell, or transfer assets	1/15
	2.3 Access to and decisions about credit	Access to and participation in decision-making concerning credit	1/15
3. Income	3.1 Control over use of income	Sole or joint control over income and expenditures	1/5
4. Leadership	4.1 Group member	Whether respondent is an active member in at least one economic or social group	1/10
	4.2 Speaking in public	Whether the respondent is comfortable speaking in public concerning issues relevant to oneself or one’s community	1/10
5. Time	5.1 Workload	Allocation of time to productive and domestic tasks	1/10
	5.2 Leisure	Satisfaction with time for leisure activities	1/10

Source: (Alkire et al., 2013).

Unlike other women’s empowerment measures based on women-only interviews, WEAI uses survey data from the self-identified primary male and female adult decision-makers in the same household. Relative empowerment is captured in GPI, which reflects women’s achievements in the five domains relative to the men in their households. Households are classified as having gender parity if either the woman is empowered (her empowerment score is 80 percent or higher) or her score is greater than or equal to the empowerment score of the male decision-maker in her household.

Like the WEAI, the 5DE and GPI are reported as aggregate indices. The values of these indices range from 0 to 1, where higher values reflect greater empowerment. The overall WEAI is a weighted average of 5DE and GPI, with weights 0.9 and 0.1, respectively. It is also a useful headline indicator, similar to the Foster-Greer-Thorbecke (FGT) indices used to track overall poverty trends. Like the FGT indices, the WEAI is decomposable, allowing us to disaggregate the 5DE achievements by domain and by indicator to identify the specific areas contributing the most to both women’s and men’s disempowerment. Although we could analyze the 10 binary subdomain indicators that comprise the 5DE, the raw count data underlying the binary indicators are better suited to detailed analyses because they have more variability for assessing the relationship between dimensions of empowerment and different nutritional outcomes. Thus, for our household- and individual-level analysis, we use the aggregate women’s empowerment score, based on the five domains of empowerment in agriculture, 10 raw count subdomain indicators on which the 5DE is based, and the gender gap in empowerment or the difference between the empowerment scores of the primary male and female decision-makers. We call the gender gap in empowerment at the household level the intrahousehold inequality score (IIS); this differs from the GPI, which is an aggregate (country- or region-level) score. Table 2 presents definitions of the empowerment indicators and the nutritional outcome indicators at the household, woman, and child levels used in this study.

**Table 2:** Definitions of empowerment, household, woman, and child outcome variables.

**Indicator**	**Definition of Indicator**
**Empowerment**	
*Empowerment*	
Empowerment score	Weighted average of achievements in the 10 indicators if the female respondent is disempowered, = 1 if she is empowered. Censored empowerment scores are used for consistency with the construction of the WEAI and other Alkire-Foster indices.
*Intrahousehold inequality*	
Intrahousehold inequality score	Difference in the male and female empowerment scores, = 0 if the female respondent is empowered.
*Subdomain indicators*	
Input in productive decisions	Number of domains an individual has some input in decisions or feels can make decisions
Autonomy in production	Number of domains an individual has autonomy in (Relative Autonomy Index >1)
Ownership of assets	Number of agricultural assets owned (sole or joint) by an individual
Purchase, sale or transfer of assets	Number of agricultural assets an individual can decide to buy, sell, transfer (sole or joint)
Access to and decisions on credit	Number of decisions over credit (sole or joint) in the last 12 months
Control over use of income	Number of income decisions (sole or joint) in the last 12 months
Group membership	Number of groups individual is member of (0–11). If the individual reports no groups in his/her community, he/she is considered a member of 0 groups.
Speaking in public	Number of contexts an individual is comfortable speaking in public (This indicator was not collected in Cambodia.)
Workload	Number of hours worked per day by individual (Mozambique: This is a binary indicator for excessive workload defined as working more than 10.5 h per day.)
Leisure	1–10 score indicating an individual’s satisfaction with time for leisure
**Household outcome**	
Household dietary diversity score	Number of food groups consumed in the last 24 h out of 9: (1) starchy staples; (2) green leafy vegetables; (3) other vitamin-A rich fruits and vegetables; (4) other fruits and vegetables; (5) organ meat; (6) meat and fish; (7) eggs; (8) legumes and nuts; (9) milk and milk products. (Bangladesh: The recall period is the last seven days instead of the last 24 h.)
**Women’s outcomes**	
Women’s dietary diversity score (*15*–*49 years old)*	Number of food groups consumed based on 24-h recall out of 9: (1) starchy staples; (2) green leafy vegetables; (3) other vitamin-A rich fruits and vegetables; (4) other fruits and vegetables; (5) organ meat; (6) meat and fish; (7) eggs; (8) legumes and nuts; (9) milk and milk products.
Women’s BMI (*15*–*49 years old)*	Log BMI form used. Defined as the ratio of weight (in kilograms) to the square of height (in meters).. Pregnant women are excluded from the BMI estimation sample for all countries.
**Children’s outcomes**	
Exclusive breastfeeding (*children 0 – 6 months)*	A child aged zero to six months is defined as exclusively breastfed if he or she did not consume any other liquids or foods other than breast milk in the preceding 24 h.
Children’s dietary diversity score (*children aged 6*–*23 mo)*	The number of food groups consumed in the last 24 h out of seven food groups, which include (1) grains, roots, and tubers; (2) legumes and nuts; (3) dairy products; (4) flesh foods; (5) eggs; (6) vitamin-A-rich fruits and vegetables; and (7) other fruits and vegetables.
HAZ (*children 0 – 23 months)*	Height-for-age Z-score
WHZ (*children 0 – 23 months)*	Weight-for-height Z-score

## 3 Data and context

We use data from the Feed the Future population-based surveys in Bangladesh (2011), Cambodia (2012), Ghana (2012), Mozambique (2012–2013), and Tanzania (2016), and one survey from the impact evaluation of Suaahara (2012), a nutrition intervention in Nepal. Although data to compute the WEAI was collected for 19 countries, most data sets do not have household- or individual-level data on nutrition-related outcomes that can be matched directly to individual WEAI data. We focus on the six countries for which individual-level data on both WEAI and nutrition are available; see International Food Policy Research Institute IFPRI, 2020.

The countries included in our study are lower- to higher-middle income countries, with gross national income (GNI) per capita in 2018 ranging from 1300 USD in Mozambique to 4650 USD in Ghana (Table 3). Agriculture, while important in these countries, accounts for less than a third of GDP in terms of value added, and a sizeable portion of the rural population lives in poverty, ranging from a fifth (Cambodia, 20.8%) to more than half (Mozambique, 56.9%). Although the reliability of statistics on the female share of employment in agriculture has been questioned (Doss, 2014), females account for a significant portion of total employment in agriculture; ranging from 43% to 60% in the countries we analyze. Conversely, agriculture is an important sector for females in terms of employment; female employment in agriculture as a share of total female employment ranges from 30.4% to 81.8%.

**Table 3:** Characteristics of study countries and estimation sample.

	South and Southeast Asia			Africa		
	Bangladesh	Nepal	Cambodia	Ghana	Mozambique	Tanzania
*Country characteristics*						
Gross national income per capita based on purchasing power parity (current international $) (2018)	4560	3090	4060	4650	1300	3160
Agriculture, value added, as share of GDP (2018) (%)	13.1	25.0	22.0	19.7^a^	21.4	28.7^a^
Proportion of rural population in poverty (%)	35.2^b^	27.4^b^	20.8^c^	37.9^c^	56.9^d^	33.3^e^
Female employment in agriculture as share of total employment in agriculture (%)	45.1^a^	60.6^d^	46.4^c^	43.4^f^	not available	not available
Female employment in agriculture as share of total female employment (2018) (%)	59.4	80.1	30.4	26.4	81.8	69.4
*Selected characteristics of estimation sample*						
Survey year	2011–2012	2012	2012	2012	2012–2013	2016
No. of households in estimation sample	3674	2937	1633	1567	910	158
No. of dual-adult households	3865	1095	1660	1627	932	185
No. of children 0–6 months	535	313	74	183	n.a.	n.a.
No. of children 6–23 months	767	1,388	374	504	353	n.a.

Source of country statistics: (Food and Agriculture Organization of the United Nations, 2019, The World Bank, n.d.-a, The World Bank, n.d.-b, The World Bank, n.d.-c, The World Bank, n.d.-d), various years ^a^2017 ^b^2010 ^c^2012 ^d^2008 ^e^2011 ^f^2015, table adapted from (Komatsu et al., 2018)*.*

Sources of survey data: Bangladesh Integrated Household Survey (2011) for Bangladesh; baseline survey of Suaahara project for Nepal (2012); and Feed the Future baseline surveys for Cambodia (2012), Ghana (2012), Mozambique (2012–2013), and Tanzani (2016). The number of dual-adult households may not be the same as those in the estimation sample owing to missing data for some variables.

The number of observations from each country varies owing to the sample size of the survey itself, the different units of observation (household, woman, child), and the degree to which data were complete or nonmissing for the dependent variables, the WEAI, and the other regressors.

Appendix Table 1 presents characteristics of the household and the respondent woman (also referred to as the primary woman) while Appendix Table 2 presents child-level characteristics, both from the estimation samples. Data on exclusive breastfeeding and anthropometry are not available for Mozambique, and child-level outcomes are not available for Tanzania.

Except for Nepal, with high rates of male outmigration, the majority of sample households are dual-adult households with an adult male and an adult female present. Women’s primary schooling completion ranges from 7.45% in Ghana to close to 80% in the Tanzania sample, which is not representative of the rural population; the age of the primary woman ranges from 37.6 years in Bangladesh to 45.6 in Ghana.^2^ Household sizes range from 4.8 in Bangladesh to 6.38 in Ghana. Households have limited dietary diversity. In the estimation sample, excluding Bangladesh, households consumed between 3 and 4 out of 9 possible food groups in the past 24 h. The higher dietary diversity in Bangladesh (8.5 food groups out of 9) may be attributable to using a longer seven-day recall period. The primary female’s dietary diversity score mirrors that of her household; although the Bangladesh household score and the women’s dietary diversity score are not comparable owing to the different recall periods. The average woman’s BMI is within normal range.

Compliance with recommended infant and young child feeding (IYCF) practices varies across our six-country sample (Appendix Table 2). Rates of exclusive breastfeeding range from 18% in Bangladesh to 78% in Cambodia. Children age 6–23 months do not have diverse diets. Thirty-two to 38% of children age 0–23 months are stunted, across our study sample, and 11–17 percent are wasted.

## 4 Methods

We adapt the methodology of Malapit and Quisumbing, 2015, Malapit et al., 2015, Sraboni et al., 2014 and estimate a pooled regression using variables available in all six countries.

Because the WEAI captures agency within the agricultural sector, we restrict the sample to rural households engaged in agriculture. We include both dual-headed and female-headed households in the analysis, as results restricted to dual headed households were similar to those using the whole sample. We restrict the sample to dual-headed households in regressions using the intrahousehold inequality score (IIS), which is the difference between the male and the female empowerment score.

We estimate the following equations using ordinary least squares:

For the household-level outcome:

(1) $$N_{h}=a_{0}+a_{1}empowerment+a_{2}I+a_{3}H+a_{4}C+\varepsilon ,$$

For woman-level outcomes:

(2) $$N_{w}=b_{0}+b_{1}empowerment+b_{2}I+b_{3}H+b_{4}C+\varepsilon ,$$

where ***N_h_*** is a vector of nutritional outcomes at the household level (such as household dietary diversity) and ***N_w_*** is a vector of woman-level nutritional outcomes (women’s dietary diversity and BMI); *empowerment* is a measure of empowerment derived from the WEAI or one of its component indicators; ***I*** is a vector of individual characteristics; ***H*** is a vector of household characteristics; ***C*** is a vector of country fixed effects, *a_1_*, ***a_2,_ a_3_***, ***a_4_***, *b_1_*, ***b_2,_ b_3_***, and ***b_4_*** are the parameters to be estimated; and *ε* is an error term. Regressions are estimated using weights, with each country weighted equally (household (or women or child) weight * 1/6).^3^

To examine the association between the subdomain WEAI indicators and the nutritional outcome, we run separate regressions on each of the 10 WEAI indicators with household and individual level controls to avoid collinearity among the indicators. The coefficients of interest are *a*_*1*_ and *b*_*1*_, which capture how the primary female’s empowerment is correlated with the nutritional outcome of either the household or primary female member, controlling for a common set of observable household characteristics (dual adult household, number of male/female household members who completed primary school, whether the primary woman completed primary school, age and age squared of the primary woman, household size, dependency ratio, household wealth quintile, geographic region) in (1) and both household and individual characteristics (whether the primary woman is literate, age, and age squared) in (2).^4^ Country fixed effects capture country-level unobservables. We also report the decomposition of the mean marginal contributions of regressor variables to the explained variance (measured by R2) using Shapley values (Huettner and Sunder, 2012).

In the child regressions (3), we control for the child’s age and sex and the mother’s age, height, and primary school completion, in addition to the household characteristics and country fixed effects described above. Estimating a pooled regression with both boys and girls assumes that women’s empowerment influences child nutritional outcomes in the same way for both sexes. To test whether women’s empowerment has a differential impact on children by sex, we include a dummy variable for the sex of the child (=1 if the child is a girl) and interact this dummy variable with the empowerment variable.

The resulting equation for child level outcomes is:

(3) $$N_{c}=c_{0}+c_{1}empowerment+c_{2}female+cb_{3}\left(empowerment\times female\right)+c_{4}I+c_{5}H+c_{6}C+\nu$$

where *c_1_*, *c_2_*, *c_3_*, ***c****_4_*, ***c****_5_*, and ***c_6_*** are the parameters to be estimated and *ν* is an error term. For boys, the relationship between women’s empowerment and children’s dietary quality, for instance, is given by *c_1_*. For girls, the relationship is given by (*c_1_ + c_3_*). If *c_3_*, the coefficient on the interaction term between empowerment and the female dummy, is significantly different from zero, this suggests that women’s empowerment has differential effects on boys and girls.

Empowerment measures may also be affected by the same factors that influence nutritional outcomes. However, because we have not found credible instruments for these indicators, we treat our estimated coefficients as indicative of associations rather than causation.

Our analysis aims to identify the dimensions of women’s empowerment that are more strongly associated with household, maternal, and child nutrition outcomes. Although these dimensions will vary across countries owing to the culture- and context-specific nature of gender dynamics, our pooled regression will enable us to identify factors that may operate across contexts. The Shapley values from these regressions also help us assess the relative contributions of these factors. The empowerment measures used in the regression analysis are operationalized in Table 2. The subdomain empowerment measures are count indicators and differ from the binary indicators used to construct the WEAI, allowing us to retain the variation in the underlying data without using the adequacy cutoffs used in the WEAI to define achievement.

## 5 Results

### 5.1 Women’s empowerment, intrahousehold inequality, and nutritional outcomes

**Fig. 1:**
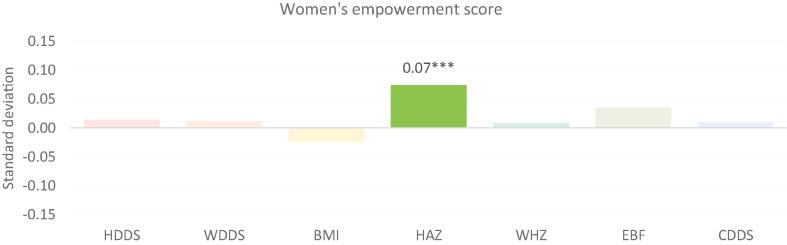
Women’s empowerment score and nutritional outcomes: Standardized coefficients. Notes: * p < 0.10, ** p < 0.05, *** p < 0.01. Solid colors depict statistically significant coefficients with standardized coefficients reported. Legend: HDDS: household dietary diversity score; WDDS: women’s dietary diversity score; BMI: body mass index (women’s); HAZ: height-for-age Z-score; WHZ: weight-for-height Z score; EBF: exclusive breastfeeding; CDDS: child’s dietary diversity score. HDDS includes 5,892 households from Bangladesh, Ghana and Tanzania. WDDS includes 11,276 women from Bangladesh, Cambodia, Nepal, Ghana, Mozambique and Tanzania. BMI includes 9390 women from Bangladesh, Cambodia, Nepal, Ghana, and Tanzania. EBF includes 902 children 0–6 months from Bangladesh, Cambodia, Nepal and Ghana. CDDS includes 2237 children 6–23 months from Bangladesh, Cambodia, Nepal, Ghana, and Mozambique. HAZ includes 2483 children 0–23 months from Bangladesh, Cambodia, Nepal and Ghana. WHZ includes 2438 children 0–23 months from Bangladesh, Cambodia, Nepal and Ghana.

**Fig. 2:**
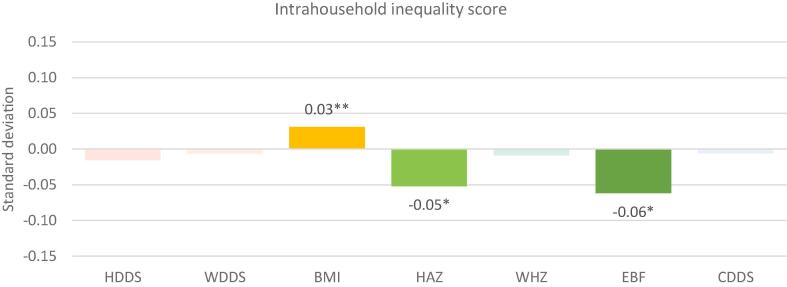
Intrahousehold inequality score and nutritional outcomes: Standardized coefficients. Notes: * p < 0.10, ** p < 0.05, *** p < 0.01. Solid colors depict statistically significant coefficients with standardized coefficients reported. Legend: HDDS: household dietary diversity score; WDDS: women’s dietary diversity score; BMI: body mass index (women’s); HAZ: height-for-age Z-score; WHZ: weight-for-height Z score; EBF: exclusive breastfeeding; CDDS: child’s dietary diversity score. HDDS includes 4976 households from Bangladesh, Ghana and Tanzania. WDDS includes 8797 women from Bangladesh, Cambodia, Nepal, Ghana, Mozambique and Tanzania. BMI includes 7331 women from Bangladesh, Cambodia, Nepal, Ghana and Tanzania. EBF includes 730 children 0–6 months from Bangladesh, Cambodia, Nepal and Ghana. CDDS includes 1628 children 6–23 months from Bangladesh, Cambodia, Nepal, Ghana, and Mozambique. HAZ includes 1817 children 0–23 months from Bangladesh, Cambodia, Nepal and Ghana. WHZ includes 1782 children 0–23 months from Bangladesh, Cambodia, Nepal and Ghana.

Fig. 1, Fig. 2 present the results from regressions of the household dietary diversity score (HDDS), women’s dietary diversity score (WDDS) and body mass index (BMI), exclusive breastfeeding (EBF), child dietary diversity score (CDDS), and child anthropometrics (HAZ and weight-for-height z-score (WHZ)) on measures of empowerment. Fig. 1 presents standardized coefficients using the women’s empowerment score based on 5DE while Fig. 2 is based on the intrahousehold inequality score. The standardized coefficients, defined as the number of standard deviations in the outcome variable that are associated with a 1.0-SD change in the empowerment variable, enable us to compare outcomes that are measured using different units. In all the figures, statistically significant associations are highlighted in brighter colors for emphasis and labeled with asterisks that correspond with the level of significance (*p < 0.10, **p < 0.05, ***p < 0.01).

The most striking result is the lack of significant association between the overall empowerment score and the intrahousehold inequality score and most of the nutritional outcomes. The only significant associations are those of the women’s empowerment score and child HAZ (positive, and in the expected direction), and of the intrahousehold inequality score with women’s BMI (positive, implying that higher intrahousehold inequality increases BMI) and EBF and HAZ (both negative, meaning that higher inequality reduces the likelihood of EBF and is associated with lower HAZ). When we analyze the data separately by country, we find that overall women’s empowerment scores are more important in relation to nutritional outcomes in the South Asian countries in our sample compared to the African ones, and that higher women’s empowerment scores are associated with better nutritional outcomes, particularly for children (see Appendix Tables 8 and 9). The strong associations in South Asia are consistent with Carlson et al. (2015), who attribute the consistent positive associations between women’s autonomy and child nutritional outcomes in South Asia to the generally lower rates of women’s autonomy in this region compared to other regions. Moreover, where significant, in our country-by-country analysis, greater equality between the primary man and woman within the same household was associated with better nutritional outcomes. In this paper, both the specification (using pooled regressions with country fixed effects) and the nature of WEAI as an aggregate index may have obscured the influence of gender norms that is evident in the country-by-country analysis.

### 5.2 Tradeoffs among dimensions of empowerment and nutrition outcomes

**Fig. 3:**
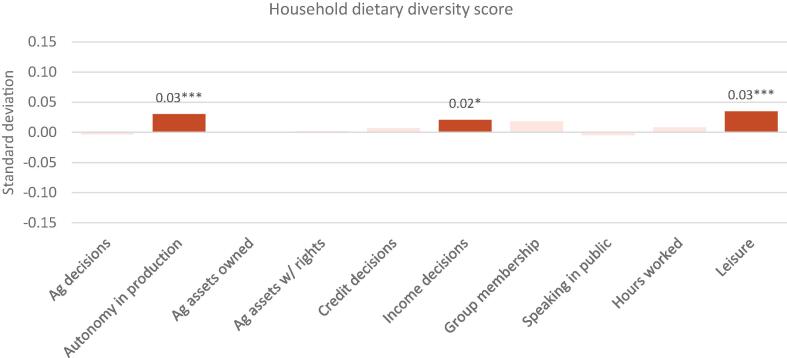
WEAI indicators and household dietary diversity score: Standardized coefficients. Notes: * p < 0.10, ** p < 0.05, *** p < 0.01. Solid colors depict statistically significant coefficients with standardized coefficients reported. HDDS includes 5982 household from Bangladesh, Ghana and Tanzania.

**Fig. 4:**
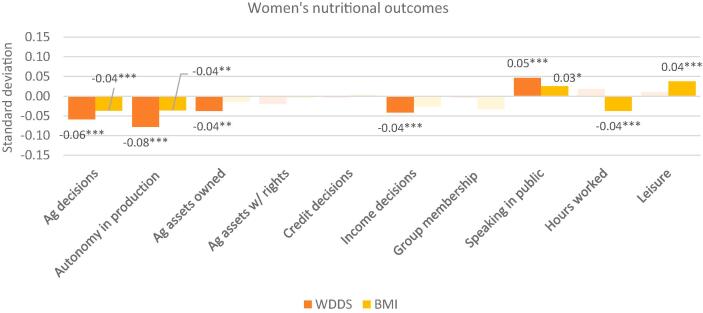
WEAI indicators and women’s dietary diversity score and body mass index: Standardized coefficients. Notes: * p < 0.10, ** p < 0.05, *** p < 0.01. Solid colors depict statistically significant coefficients with standardized coefficients reported. Legend: WDDS: women’s dietary diversity score; BMI: body mass index (women’s). WDDS includes 11,276 women from Bangladesh, Cambodia, Nepal, Ghana, Mozambique and Tanzania. BMI includes 9390 women from Bangladesh, Cambodia, Nepal, Ghana and Tanzania.

**Fig. 5:**
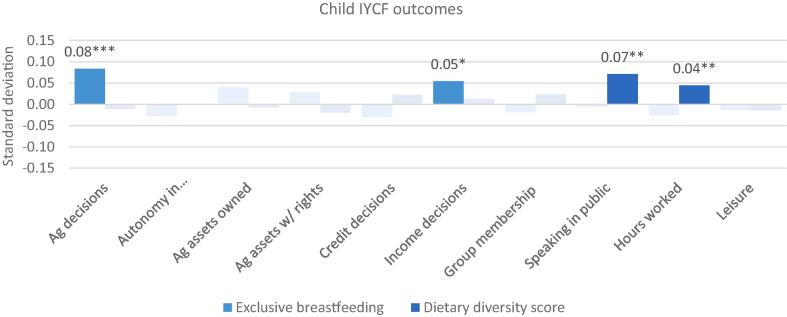
WEAI indicators and infant and young child feeding outcomes. Notes: * p < 0.10, ** p < 0.05, *** p < 0.01. Solid colors depict statistically significant coefficients with standardized coefficients reported. EBF includes 902 children 0–6 months from Bangladesh, Cambodia, Nepal and Ghana. CDDS includes 2237 children 6–23 months from Bangladesh, Cambodia, Nepal, Ghana, and Mozambique.

**Fig. 6:**
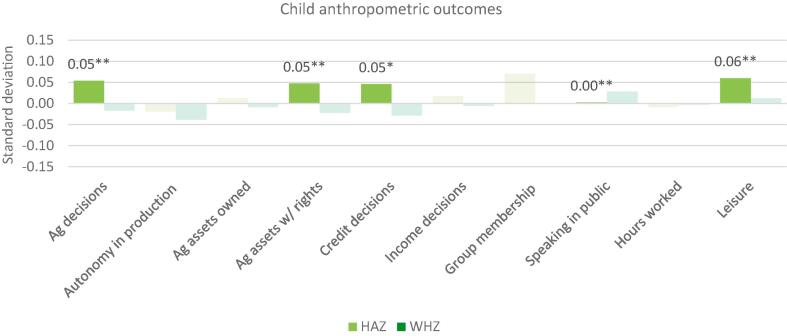
WEAI indicators and child anthropometric outcomes. Notes: * p < 0.10, ** p < 0.05, *** p < 0.01. Solid colors depict statistically significant coefficients with standardized coefficients reported. Legend: HAZ: height-for-age Z-score; WHZ: weight-for-height Z score. HAZ includes 2483 children 0–23 months from Bangladesh, Cambodia, Nepal and Ghana. WHZ includes 2438 children 0–23 months from Bangladesh, Cambodia, Nepal and Ghana.

Because changes in the component subdomain indicators of the WEAI may offset each other, indicating possible tradeoffs between various dimensions of empowerment, the relationship between the indicators and the WEAI is not necessarily monotonic. For example, increased participation in agricultural production or in groups may increase workload. The offsetting effects may explain the lack of statistically significant relationships between the aggregate score and nutritional outcomes, necessitating the “unpacking” of the results by indicator. Thus, we estimate a third version of the regressions, Model 3, which is run separately on each of the 10 WEAI subdomain indicators (expressed as count variables) with the same household, individual, and country controls as Models 1 and 2. Estimated coefficients for all 10 indicators, with standard errors in parentheses, are presented in the same table for compactness in Appendix Tables 3–6; standardized coefficients are presented in Fig. 3, Fig. 4 for household and maternal outcomes, respectively, Fig. 5 for IYCF outcomes, and Fig. 6 for child anthropometric outcomes.

#### 5.2.1 Household dietary diversity

Fig. 3 presents standardized coefficients for the ten WEAI subdomain indicators and the household dietary diversity score, estimated by pooling observations across Bangladesh, Ghana, and Tanzania. Autonomy in production, control over income decisions, and satisfaction with time spent or leisure are all positively associated with HDDS, suggesting that tradeoffs do not exist among these dimensions of empowerment, at least so far as household outcomes are concerned.

#### 5.2.2 Women’s outcomes

Fig. 4 graphs the standardized coefficients for the 10 WEAI subdomain indicators and women’s dietary diversity score (WDDS) and BMI. Unlike the results for HDDS, we find a more nuanced pattern of associations compared with those obtained using the aggregate scores, indicating that some dimensions of women’s empowerment may be negatively associated with better nutritional outcomes for women. A greater number of agricultural decisions, greater autonomy in production, a greater number of agricultural assets owned, and a greater number of income decisions are associated with lower WDDS, while greater confidence in speaking in public is associated with higher WDDS. A greater number of agricultural decisions, more autonomy in production, and a higher number of hours worked are associated with lower BMI, while comfort with speaking in public and satisfaction with leisure are associated with higher BMI. Most of the subdomain indicators that are associated with lower BMI are those that are typically linked with greater direct involvement in agriculture. Potential tradeoffs between empowerment and nutritional outcomes may arise because increased involvement in agriculture, which increases women’s empowerment score, also increases women’s workload. Time use (workload + leisure) associations show the most consistent associations with nutrition outcomes across countries in our country-by-country analysis. In the country-specific analysis, higher workloads are associated with higher WDDS in Mozambique and Tanzania, and with lower women’s BMI in Bangladesh, Nepal, and Ghana. In Cambodia and Ghana, higher workload is associated with worse child anthropometric outcomes. Although BMI has been useful in population-based studies partly because of ease and economy of data collection and its wide acceptance in defining specific categories of body mass as a health issue, it has been criticized as a poor indicator of percent of body fat (Nuttall, 2015). We therefore interpret our results as suggestive of the associations between agricultural work, energy expenditure, and women’s body mass, and not as indicative of women’s health in general.

#### 5.2.3 Child outcomes

Fig. 5 presents standardized coefficients for the 10 WEAI subdomain indicators and two measures of IYCF: exclusive breastfeeding (EBF) and the children’s dietary diversity score (CDDS). Unlike the results with the empowerment aggregates, some significant associations emerge. A higher number of agricultural decisions is associated with a higher likelihood that the index child is exclusively breastfed and greater comfort speaking in public and more hours worked are linked with higher CDDS. This suggests that no tradeoffs exist among empowerment indicators with respect to these two IYCF outcomes.

Neither do tradeoffs exist with respect to child anthropometry. Higher HAZ, an indicator of long-term nutritional status, is associated with more agricultural decisions, a higher number of agricultural assets with rights, a higher number of credit decisions, and greater satisfaction with leisure (Fig. 6). This result is consistent with the positive association of HAZ with the aggregate women’s empowerment score.

### 5.3 How important is women’s empowerment relative to other correlates of nutrition?

Although some of the associations of women’s empowerment with nutritional outcomes are significant, are they meaningful? Do women’s empowerment indicators account for a high proportion of the variation in nutritional outcomes? We answer this in two ways. First, we assess the magnitude of the estimated coefficients where they are significantly different from zero. Second, we calculate Shapley values, which decompose the explained variance of the regressions (measured by R^2^) into contributions from particular variables or groups of variables to the overall regression model R^2^ (Huettner and Sunder 2012, cited in Sheahan and Barrett 2017).

The women’s empowerment score is significantly different from zero only in the HAZ regression and the intrahousehold inequality score is significant in the BMI, EBF, and HAZ regressions (Table 5, Table 6, Table 7). These estimated coefficients are relatively small in magnitude in the pooled regressions with country fixed effects. A 10 percentage point (pp) increase in the women’s empowerment score is associated with an increase in child HAZ of 0.05, while a 10 pp decrease in the intrahousehold inequality score is associated with a 0.04 increase in child HAZ and a one percent increase in the likelihood that a child is exclusively breastfed. However, country-level regressions show that the associations may be stronger for specific countries; in the pooled regressions, these intra-country variations are swamped by the country fixed effects (see the discussion below in the Shapley decompositions). For example, the Cambodia country-level regression shows that a 10 pp increase in the empowerment score (10 pp decrease in the intrahousehold inequality score) is associated with a 16 percent increase in the likelihood that a child will be exclusively breastfed. The country-level regressions also show significant associations between the empowerment score and HAZ for Nepal, and between the empowerment score and intrahousehold inequality score for CDDS in Ghana, but with small magnitudes similar to the pooled results (see Appendix Table 9 for the country-level results).

Consistent with the magnitudes of association with some child outcomes, women’s empowerment indicators (both empowerment score and intrahousehold inequality) are statistically indistinguishable from zero; Shapley decompositions show that the women’s empowerment indicators do not account for a sizeable proportion of the variation in HDDS (Table 4). The greatest contribution to HDDS is from household wealth, which accounts for close to 50 percent of the variance in HDDS for both empowerment measures, country fixed effects (28–30%), and the number of females completing primary school in the household (9–10%). The respondent woman’s primary school completion contributes about 5 percent to the variation in HDDS, but the contribution of either measure of empowerment is less than one percent.

**Table 4:** Women’s empowerment and household dietary diversity score.

Variables	Model 1: Empowerment score				Model 2: Intrahousehold inequality score			
	Coeff.	Sig.	SE	Shapley	Coeff.	Sig.	SE	Shapley
	svy^a^	svy^a^	svy^a^	rego^b^	svy^a^	svy^a^	svy^a^	rego^b^
Empowerment regressor	0.122		0.097	0.282	−0.155		0.125	0.526
***Primary female respondent characteristics***								
Age	−0.012		0.016	0.106	−0.011		0.018	0.157
Age squared	0.000		0.000	0.010	0.000		0.000	0.123
Completed primary school	0.163	**	0.076	5.339	0.116		0.085	4.737
***Household characteristics***								
Dual headed	0.118	*	0.067	0.319	–	–	–	0.000
Primary respondent age	−0.007		0.014	0.150	−0.006		0.016	0.222
Primary respondent age squared	−0.000		0.000	0.136	−0.000		0.000	0.191
# of males completed primary school	0.035		0.034	3.773	0.037		0.037	4.257
# of females completed primary school	0.204	***	0.046	9.378	0.249	***	0.053	10.163
HH size	0.149	***	0.014	1.909	0.146	***	0.016	1.822
Dependency ratio	0.161	***	0.032	0.664	0.185	***	0.041	0.988
Wealth quintile 2	0.927	***	0.069	3.220	0.928	***	0.075	3.246
Wealth quintile 3	1.487	***	0.071	3.711	1.509	***	0.078	3.942
Wealth quintile 4	2.173	***	0.071	11.588	2.165	***	0.078	11.591
Wealth quintile 5	2.925	***	0.075	29.379	2.950	***	0.083	30.016
***Country dummy variables***								
Cambodia	n/a				n/a			
Nepal	n/a				n/a			
Ghana	−2.042	***	0.067	29.490	−2.011	***	0.077	27.744
Mozambique	n/a				n/a			
Tanzania	0.415	***	0.136	0.456	0.191		0.161	0.276

Notes: n = 5892 households in Bangladesh, Ghana and Tanzania. Household dietary diversity score not available in Cambodia, Nepal or Mozambique.

a Svy estimates use household level weights (pweights).

b Household level weights are not used for the reported Shapley coefficient created by using the “rego” command in Stata.

* p < 0.1.

** p < 0.05.

*** p < 0.01.

**Table 5:** Women’s empowerment, women’s dietary diversity score and body mass index.

Variables	Women’s Dietary Diversity Score						Body mass index					
	Model 1: Women’s empowerment score			Model 2: Intrahousehold inequality score			Model 1: Women’s empowerment score			Model 2: Intrahousehold inequality score		
	Coeff.	SE	Shapley	Coeff.	SE	Shapley	Coeff.	SE	Shapley	Coeff.	SE	Shapley
	svy^a^	svy^a^	rego^b^	svy^a^	svy^a^	rego^b^	svy^a^	svy^a^	rego^b^	svy^a^	svy^a^	rego^b^
Empowerment regressor	0.075	0.082	0.629	−0.052	0.100	0.243	−0.015	0.009	2.878	0.023**	0.012	0.596
***Primary female respondent characteristics***												
Age	0.013	0.013	0.275	0.024	0.015	0.433	0.003**	0.001	2.621	0.004***	0.002	4.381
Age squared	−0.000	0.000	0.375	−0.000**	0.000	0.676	−0.000***	0.000	2.456	−0.000***	0.000	4.256
Completed primary school	−0.069	0.065	3.176	−0.091	0.075	2.381	0.019***	0.006	5.156	0.019***	0.007	3.625
***Household characteristics***												
Dual headed	0.020	0.053	0.440	–	–	–	−0.006	0.005	0.238	–	–	–
Primary respondent age	−0.025**	0.011	0.158	−0.029**	0.013	0.160	0.002*	0.001	1.520	0.002*	0.001	2.714
Primary respondent age squared	0.000**	0.000	0.134	0.000**	0.000	0.148	−0.000*	0.000	1.502	−0.000	0.000	2.577
# of males completed primary school	0.067***	0.025	3.331	0.060**	0.027	3.338	0.013***	0.003	4.820	0.014***	0.003	6.923
# of females completed primary school	0.120***	0.031	8.285	0.139***	0.037	7.833	−0.002	0.003	0.594	−0.004	0.003	0.562
HH size	0.025***	0.010	0.434	0.013	0.010	0.467	−0.001*	0.001	1.067	−0.000	0.001	1.126
Dependency ratio	0.173***	0.027	1.746	0.215***	0.033	1.688	0.008***	0.003	0.835	0.007*	0.004	0.581
Wealth quintile 2	0.313***	0.062	3.961	0.296***	0.070	4.824	0.026***	0.006	1.311	0.019***	0.006	1.389
Wealth quintile 3	0.564***	0.061	4.089	0.596***	0.068	4.365	0.025***	0.005	1.405	0.021***	0.006	1.406
Wealth quintile 4	0.874***	0.062	19.175	0.894***	0.071	20.196	0.044***	0.006	3.958	0.041***	0.006	3.648
Wealth quintile 5	1.145***	0.064	37.427	1.180***	0.075	37.473	0.072***	0.006	23.633	0.067***	0.007	22.888
***Country dummy variables***												
Cambodia	−0.014	0.067	1.595	0.073	0.069	2.792	0.029***	0.006	8.784	0.031***	0.006	12.281
Nepal	−0.160***	0.047	0.902	−0.100*	0.053	0.348	−0.021***	0.005	7.407	−0.013**	0.006	3.825
Ghana	−0.132**	0.062	0.886	−0.157**	0.070	1.312	0.051***	0.006	23.617	0.048***	0.007	21.352
Mozambique	−0.585***	0.071	10.503	−0.580***	0.081	8.999	n/a	n/a	n/a	n/a	n/a	n/a
Tanzania	0.632***	0.129	2.479	0.676***	0.152	2.423	0.067***	0.017	6.200	0.068***	0.019	5.870

Notes: Women’s dietary diversity score: n = 11,276 women 15–49 in Bangladesh, Cambodia, Nepal, Ghana, Mozambique and Tanzania. Women’s BMI: n = 9390 women 15–49 in Bangladesh, Cambodia, Nepal, Ghana, and Tanzania.

a Svy estimates use women level (pweights).

b Women level weights are not used for the reported Shapley coefficient created by using the “rego” command in Stata.

* p < 0.1.

** p < 0.05.

*** p < 0.01.

Similarly, household wealth contributes between 65 and 67 percent of the variance in women’s dietary diversity in regressions using the empowerment score and the intrahousehold inequality score, followed by country fixed effects (around 16%), the number of women completing primary school in the household (around 8%), and whether the woman herself finished primary school (3.2–5.2%). Women’s empowerment, whether measured using the empowerment score or the intrahousehold inequality score, has a negligible contribution to the variation in WDDS. Similarly, household wealth (29–67%) and country fixed effects (43–46%) are the major contributors to the variation in BMI, but the woman respondent’s own characteristics (age, age squared, and primary school completion), which contribute 10–12 percent of the variation, are also important. The women’s empowerment score contributes 5 percent to the variation in BMI, but the estimated coefficient is not significantly different from zero.

Similarly, although the empowerment indicators are of the expected sign in the IYCF regressions (positive for the empowerment score, negative for intrahousehold inequality), they contribute a small proportion of the variation in EBF or CDDS (Table 6). The single most important factor affecting these two outcomes is child age. If we include the quadratic term in child age, the contribution of age and age squared together account for 34–86 percent of the variation in IYCF practices.

**Table 6:** Women’s empowerment and infant and young child feeding practices.

Variables	Exclusive breastfeeding						Child dietary diversity score					
	Model 1: Women’s empowerment score			Model 2: Intrahousehold inequality score			Model 1: Women’s empowerment score			Model 2: Intrahousehold inequality score		
	Coeff.	SE	Shapley	Coeff.	SE	Shapley	Coeff.	SE	Shapley	Coeff.	SE	Shapley
	svy^a^	svy^a^	rego^b^	svy^a^	svy^a^	rego^b^	svy^a^	svy^a^	rego^b^	svy^a^	svy^a^	rego^b^
(1.a) Empowerment regressor	0.069	0.067	0.825	−0.133	0.087	0.453	0.401**	0.193	0.731	−0.176	0.219	0.320
(1.b) Empowerment regressor * girl	−0.008	0.100	0.176	0.040	0.117	0.197	−0.456*	0.250	0.234	0.301	0.306	0.059
Average coeff. of empowerment: (1.a) + (1.b) * % girls	0.065			−0.115*			0.180			−0.028		
p-value of F-test: (1.a) + (1.b) * % girls = 0	0.191			0.051			0.236			0.871		
***Child characteristics***												
Age	−0.004*	0.002	31.918	−0.003	0.002	29.722	0.198***	0.007	59.445	0.190***	0.007	61.419
Age squared	−0.018**	0.003	5.020	−0.015***	0.003	4.066	−0.001***	0.000	25.896	−0.001**	0.000	24.709
Girl	0.018	0.066	0.136	−0.011	0.033	0.159	0.366**	0.161	0.216	−0.006	0.100	0.197
***Mother characteristics***												
Age	0.007	0.014	0.273	0.001	0.016	0.194	−0.035	0.034	0.137	−0.035	0.042	0.157
Age squared	−0.000	0.000	0.294	0.000	0.000	0.200	0.001	0.001	0.136	0.001	0.001	0.160
Mother height	0.001	0.002	3.429	0.002	0.002	4.540	−0.006	0.005	0.227	−0.007	0.006	0.308
Completed primary	−0.023	0.051	1.256	0.013	0.055	1.034	0.149	0.101	0.316	0.038	0.118	0.143
***Household characteristics***												
Dual headed	−0.018	0.036	0.721	n/a	n/a	n/a	−0.013	0.069	0.402	n/a	n/a	n/a
Primary female completed primary school	0.025	0.046	2.109	−0.034	0.050	2.180	−0.133	0.110	0.140	−0.071	0.127	0.123
Primary respondent age	−0.012*	0.006	0.567	−0.009	0.007	0.452	0.031**	0.014	0.250	0.031*	0.017	0.192
Primary respondent age squared	0.000*	0.000	0.566	0.000	0.000	0.414	−0.000**	0.000	0.212	−0.000*	0.000	0.167
# of males completed primary school	−0.031*	0.018	0.490	−0.040**	0.020	0.736	0.082**	0.040	0.637	0.092*	0.049	0.724
# of females completed primary school	−0.018	0.025	0.712	0.009	0.029	0.364	0.121**	0.050	0.508	0.162***	0.061	0.532
HH size	0.017*	0.009	3.245	0.021**	0.010	4.794	−0.043***	0.015	0.469	−0.047**	0.019	0.585
Dependency ratio	−0.043*	0.023	0.370	−0.026	0.032	0.251	0.014	0.051	0.176	0.047	0.076	0.160
Wealth quintile 2	0.007	0.037	0.076	0.001	0.041	0.039	0.304***	0.097	0.179	0.276**	0.115	0.140
Wealth quintile 3	0.054	0.034	0.308	0.052	0.035	0.257	0.236**	0.094	0.115	0.262**	0.113	0.128
Wealth quintile 4	0.050	0.035	0.099	0.057	0.038	0.129	0.257***	0.093	0.211	0.195*	0.112	0.140
Wealth quintile 5	0.022	0.037	0.120	0.034	0.039	0.109	0.465***	0.104	1.530	0.458***	0.131	1.816
***Country dummy variables***												
Cambodia	0.821***	0.080	20.956	0.809***	0.081	24.422	1.322***	0.138	2.652	1.240***	0.145	3.342
Nepal	0.577***	0.057	14.435	0.546***	0.065	12.808	1.064***	0.091	3.952	0.967***	0.106	3.339
Ghana	0.579***	0.075	11.897	0.547***	0.087	12.480	0.973***	0.139	1.230	0.793***	0.161	1.142
Mozambique	n/a	n/a	n/a	n/a	n/a	n/a	–		–	–		–
Tanzania	n/a	n/a	n/a	n/a	n/a	n/a	n/a	n/a	n/a	n/a	n/a	n/a

Notes: Exclusive breastfeeding: n = 902 children 0–6 months in Bangladesh, Cambodia, Nepal and Ghana. Exclusive breastfeeding not available for Mozambique or Tanzania.

Child dietary diversity score: n = 2237 children 6–23 months in Bangladesh, Cambodia, Nepal, Ghana and Mozambique. Mozambique country dummy dropped due to multicollinearity. Child dietary diversity score not available for Tanzania.

a Svy estimates use child level (pweights).

b Child level weights are not used for the reported Shapley coefficient created by using the “rego” command in Stata.

* p < 0.1.

** p < 0.05.

*** p < 0.01.

Finally, despite the significant association between women’s empowerment indicators and HAZ, biological factors (child age) account for most of the variation in child HAZ and WHZ, although maternal height is also important for HAZ, accounting for 17 percent of the variance, along with country fixed effects (Table 7). Overall, it appears that the contribution of women’s empowerment to the variance in nutritional outcomes is very small relative to other correlates of nutritional status.

**Table 7:** Women’s empowerment and child anthropometry.

Variables	Height-for-age z-score						Weight-for-height z-score					
	Model 1: Women’s empowerment score			Model 2: Intrahousehold inequality score			Model 1: Women’s empowerment score			Model 2: Intrahousehold inequality score		
	Coeff.	SE	Shapley	Coeff.	SE	Shapley	Coeff.	SE	Shapley	Coeff.	SE	Shapley
	svy^a^	svy^a^	rego^b^	svy^a^	svy^a^	rego^b^	svy^a^	svy^a^	rego^b^	svy^a^	svy^a^	rego^b^
(1.a) Empowerment regressor	0.567**	0.226	1.715	−0.506	0.312	0.998	−0.023	0.204	0.975	0.007	0.267	0.714
(1.b) Empowerment regressor * girl	−0.092	0.278	0.824	0.259	0.380	0.195	0.099	0.253	0.953	−0.150	0.325	0.362
Average coeff. of empowerment: (1.a) + (1.b) * % girls	0.523***			−0.379*			0.025			−0.067		
p-value of F-test: (1.a) + (1.b) * % girls = 0	0.002			0.074			0.865			0.715		
***Child characteristics***												
Age	−0.073***	0.009	40.602	−0.073***	0.010	44.114	−0.036***	0.009	13.234	−0.035***	0.009	19.221
Age squared	−0.000	0.000	12.033	−0.000	0.000	9.257	0.001*	0.000	5.402	0.001	0.000	4.518
Girl	0.250	0.190	0.573	0.174	0.113	0.889	−0.202	0.168	1.088	−0.108	0.098	1.251
***Mother characteristics***												
Age	0.032	0.039	0.361	0.020	0.051	0.273	0.021	0.034	0.416	0.017	0.042	0.251
Age squared	−0.001	0.001	0.409	−0.000	0.001	0.289	−0.001	0.001	0.468	−0.000	0.001	0.219
Mother height	0.038***	0.006	16.704	0.037***	0.007	16.723	−0.000	0.005	1.579	−0.003	0.006	1.588
Completed primary	0.080	0.116	0.809	0.071	0.139	0.502	0.138	0.104	4.169	0.132	0.118	3.395
***Household characteristics***												
Dual headed	−0.015	0.075	0.186	n/a	n/a	n/a	0.150**	0.070	7.445	n/a	n/a	n/a
Primary female completed primary school	−0.111	0.125	0.442	−0.167	0.150	0.616	−0.017	0.105	1.428	0.000	0.123	0.891
Primary respondent age	−0.021	0.016	0.902	−0.043**	0.019	0.948	0.000	0.015	0.574	−0.005	0.018	0.858
Primary respondent age squared	0.000*	0.000	1.039	0.000***	0.000	1.278	0.000	0.000	0.545	0.000	0.000	0.933
# of males completed primary school	0.003	0.043	0.342	0.001	0.055	0.469	−0.014	0.037	2.863	0.026	0.044	2.204
# of females completed primary school	0.130**	0.056	1.247	0.165**	0.072	1.552	0.04	0.045	1.130	0.000	0.052	0.784
HH size	−0.001	0.019	0.420	0.001	0.025	0.524	−0.038***	0.014	6.600	−0.055***	0.018	11.339
Dependency ratio	−0.144**	0.063	1.063	−0.165*	0.098	0.915	0.035	0.057	0.285	0.042	0.085	0.843
Wealth quintile 2	0.143	0.118	0.301	0.076	0.147	0.229	0.184*	0.106	2.057	0.167	0.127	1.492
Wealth quintile 3	0.104	0.116	0.124	0.014	0.146	0.100	0.149	0.107	1.271	0.173	0.133	2.097
Wealth quintile 4	0.135	0.116	0.242	0.042	0.149	0.145	0.280***	0.104	5.761	0.247*	0.128	6.433
Wealth quintile 5	0.208*	0.114	0.244	0.135	0.151	0.164	0.266**	0.107	7.790	0.230*	0.132	9.356
***Country dummy variables***												
Cambodia	−0.046	0.167	0.684	0.056	0.183	0.944	0.043	0.146	2.501	−0.003	0.160	1.017
Nepal	−0.005	0.119	1.770	0.017	0.136	1.205	−0.382***	0.107	20.614	−0.290**	0.124	12.942
Ghana	0.635***	0.177	16.965	0.629***	0.208	17.672	0.267*	0.149	10.855	0.388**	0.171	17.293
Mozambique	n/a	n/a	n/a	n/a	n/a	n/a	n/a	n/a	n/a	n/a	n/a	n/a
Tanzania	n/a	n/a	n/a	n/a	n/a	n/a	n/a	n/a	n/a	n/a	n/a	n/a

Notes: Height-for-age: n = 2483 children 0–23 months in Bangladesh, Cambodia, Nepal and Ghana. Weight-for-height: n = 2438 children 0–23 months in Bangladesh, Cambodia, Nepal and Ghana.

a Svy estimates use child level (pweights).

b Child level weights are not used for the reported Shapley coefficient created by using the “rego” command in Stata.

* p < 0.1.

** p < 0.05.

*** p < 0.01.

### 5.4 Interactions with child gender

Do more empowered mothers treat sons or daughters preferentially? We test this by examining interactions of child sex with the empowerment indicator. In contrast to the country-specific results, interactions of either the empowerment score or the intrahousehold inequality score with child gender are generally insignificant in the pooled regressions, with the exception of CDDS, where women’s empowerment is differentially associated with worse outcomes for girls, albeit only weakly significant (Table 6). Regression results using the 10 WEAI subdomain indicators (Appendix Tables 5 and 6) point to a subtle pattern of gender preference. Regressions on ICYF outcomes (Appendix Table 5) show that a higher number of decisions on credit are associated with a higher likelihood that girls are exclusively breastfed, but a larger number of rights over assets is differentially associated with better dietary diversity outcomes for boys. Both interactions, however, are only weakly significant. None of the interaction terms with child gender are significant in the HAZ regressions, but a pattern of tradeoffs emerges in WHZ (Appendix Table 6). The respondent woman having greater satisfaction with leisure is associated with girls differentially having lower WHZ, whereas having greater control over income and greater comfort speaking in public are associated with higher WHZ for girls.

In the country-specific analysis, we found several instances of significant interaction effects with child gender, suggesting that women’s empowerment has differential associations with boys’ and girls’ nutritional outcomes. In Bangladesh, for example, women’s empowerment has a negative association for girls relative to boys, which may reflect the persistence of son preference because women depend on sons for old age support. This is consistent with findings of Sraboni and Quisumbing, 2018, Malapit et al., 2019. In Nepal, few differential outcomes by child’s gender were significant except that a higher number of agricultural decisions made by the mother is associated with a differential advantage for boys with respect to WAZ. In Cambodia, in contrast, a larger number of decisions made by women had differential positive associations for girls’ anthropometric outcomes compared to boys. Oddly, a higher intrahousehold inequality score is consistently associated with higher HAZ and WAZ scores for girls. These results, which contrast with the two South Asian countries, may reflect the more egalitarian nature of gender relations and the lower degree of son preference in Southeast Asia.

In Ghana, consistent with findings in Malapit and Quisumbing (2015), the interactions with the girl variable for children’s dietary diversity score were significant and positive for the intrahousehold inequality score and negative for the 5DE score. This suggests that girls consume more diverse diets in households where women are less empowered, and empowerment gaps between men and women are wider. This counter-intuitive result may be traced to the practice of feeding porridge as the primary complementary food to healthy children, whereas children who rejected the porridge or had poor appetite due to illness are more likely to be offered other foods to encourage them to eat (Davis, Tagoe-Darko, & Mukuria, 2003).^5^ In Mozambique, several significant interaction effects indicated different associations for girls and boys. Girls experienced worse IYCF outcomes compared to boys for empowerment indicators related to ownership and rights over assets, group membership, and hours worked per day. The variation in the extent to which women’s empowerment is differentially associated with child outcomes depending on gender may be traced to cultural norms and is likely to be masked in pooled regressions.

### 5.5 Robustness to aggregation method

The WEAI, as an aggregate index, uses the Alkire-Foster method with predetermined thresholds to determine adequacy and fixed weights. Are our results robust to our choice of aggregation method? Appendix Table 7 explores this using the first principal component of the 10 WEAI indicators. Similar to the results using the WEAI, the index created using principal components analysis (PCA) does not exhibit any significant associations with household dietary diversity and women’s dietary diversity but shows a statistically significant albeit small association with women’s BMI. We also find an unexpected negative correlation with child dietary diversity (and a differential effect that favors girls) and HAZ, but the Shapley values indicate that the contribution of women’s empowerment to the variance in nutritional outcomes is very small relative to other correlates of nutritional status, consistent with results using the WEAI.

## 6 Discussion and policy implications

Our overall results indicate that associations between our aggregate indicators of women’s empowerment (the women’s empowerment score and the intrahousehold inequality scores) and nutritional outcomes are weak, except for those with children’s HAZ, exclusive breastfeeding, and women’s BMI. When we analyze the subdomain indicators that comprise the WEAI, a more subtle pattern emerges. Autonomy in production, control over income decisions, and satisfaction with time spent or leisure are all positively associated with HDDS, suggesting that no tradeoffs appear with respect to household-level outcomes. Neither do tradeoffs exist among empowerment indicators with respect to the two IYCF outcomes of EBF and CDDS and with respect to child anthropometric outcomes. Higher HAZ, an indicator of long-term nutritional status, is associated with more agricultural decisions, a higher number of agricultural assets with rights, a higher number of credit decisions, and greater satisfaction with leisure.

However, for the woman’s own outcomes, tradeoffs become apparent. Indicators of greater direct involvement in agricultural production (a greater number of agricultural decisions, greater autonomy in production, a greater number of agricultural assets owned) are associated with lower WDDS, while greater confidence in speaking in public is associated with higher WDDS. Comfort with speaking in public and satisfaction with leisure are associated with higher BMI, but a higher number of work hours is associated with lower BMI. The associations between BMI and work hours, particularly for poorer women in low-BMI populations, may reflect tensions among women’s productive and reproductive roles, given time and energy constraints. These may also reflect cultural norms in which an empowered woman is viewed as one who can ensure her family’s and children’s food and nutrition security, rather than as one who takes care of her own welfare.

The tradeoffs that we uncover are consistent with the findings of the systematic reviews (Carlson et al., 2015, Pratley, 2016, Santoso et al., 2019) that not all empowerment domains are positively correlated with better nutrition. Women may work more to increase the quantity and quality of food available to their households, but longer work hours may also increase her energy expenditure, with consequences of lower maternal BMI and less time for childcare. Analysis of time use data from the WEAI in the same countries (except Tanzania) shows that women’s domestic work and cooking time are positively correlated with more diverse diets (Komatsu et al. 2018), but that effects differ according to asset poverty status, with long hours spent in agriculture more likely to be important as a source of food and income for the poor. Komatsu et al. (2018) conclude that women’s time allocation and nutrition responses to agricultural interventions are likely to vary by socioeconomic status and local context.

Son preference is a cultural phenomenon underlying our results for child anthropometric outcomes in Bangladesh, Nepal, and Ghana, and emerges in the pooled sample. In these countries, women’s empowerment is consistently associated with worse outcomes for girls; in Cambodia, although the interaction with child gender is weakly significant, women’s empowerment is associated with better outcomes for girls, reflecting the generally more egalitarian gender norms in Southeast Asia. While cultural preference for boys is deeply embedded in many cultures, programs could be designed to negate this differential – for instance, through providing a food ration or nutritional supplement specifically for girls and providing behavior-change communication to mothers and fathers that emphasizes the need to treat children equally, regardless of gender.

Carlson et al. (2015) argue that understanding which dimensions of autonomy result in the most health benefits for women and children could help focus public health interventions on improving the most important aspects of women’s autonomy as immediate goals (Carlson et al., 2015). Although the WEAI includes an autonomy measure, it relates to agricultural production, not health and nutrition outcomes. Nevertheless, our findings suggest that policymakers and program designers need to be aware of tradeoffs among different dimensions of empowerment to avoid unintended consequences, particularly for mothers of young children. This policy implication differs from that derived from previous analyses. Previously, the WEAI had been used to identify policy and programming priorities by disaggregating the contribution of each indicator to women’s disempowerment, identifying the top two or three contributors, and recommending that programs be designed to support empowerment in these specific areas (Malapit et al., 2015, Malapit and Quisumbing, 2015, Sraboni et al., 2014). The present analysis assessing all ten indicators finds that focusing on the top two or three contributors to women’s disempowerment provides little, if not potentially misguided, direction for improving nutritional outcomes at the household-, woman-, and child-levels. Considering all 10 indicators provides a fuller picture of which indicators matter for which nutritional outcomes in a given context and reveals potential tradeoffs that should be considered.

The Shapley decompositions highlight the large contribution of country fixed effects and wealth to variations in nutritional status. The large share of the variation in household and women’s dietary diversity accounted for by household wealth suggests that increasing the wealth of rural households remains an important concern. Similarly, the large proportion of the variance accounted for by country-level effects points to the importance of gender norms and country-specific institutions. Most of the variation in child outcomes comes from child age, suggesting that any intervention to improve children’s nutritional status must be age appropriate. Notably, these results may disappoint those who think empowering women will solve all nutritional problems: despite its positive association with many outcomes, it is not a cure-all. To improve nutrition of women, their households, and their children, the underlying determinants of poor nutrition need to be addressed, in addition to empowering women and improving gender equality.

These results need to be viewed in light of this study’s limitations. First, the dimensions of empowerment included in the WEAI may not include those directly related to nutrition, unlike the Women’s Empowerment in Nutrition Index (WENI) (Narayanan et al. 2019). Second, the process of aggregation may mask tradeoffs among different dimensions of nutrition, which we have tried to address by analyzing subdomain indicators. Third, women’s empowerment itself may also be affected by such variables as country context, particularly wealth, social norms, and schooling—factors that account for a large share of the variance in nutritional outcomes. Lastly, the estimated relationships between women’s empowerment measures and nutrition outcomes are associations, and do not indicate causation. Assessing the magnitude of the gender-sensitive agricultural programs on nutrition outcomes requires impact evaluations that measure both empowerment and nutrition outcomes.

Our findings suggest that we should not assume that nutritional outcomes will improve automatically in interventions that aim to empower women. This caveat is very similar to Ruel and Alderman’s (2013) sobering finding that nutrition improvements are not automatic even with programs that succeed in reducing poverty, food insecurity, and sex inequalities. They argue that, to reach their full potential, such programs need careful identification of nutrition goals and appropriate design and effective implementation of interventions to achieve them. Engaging women in nutrition-sensitive programs must therefore include interventions to protect and promote their nutritional wellbeing, physical and mental health, social status, decision making, and their overall empowerment and ability to manage their time, resources, and assets. Policymakers and program designers must be mindful of the tradeoffs women face as agricultural producers and as stewards of their household’s and their own food and nutrition security.

## CRediT authorship contribution statement

**Agnes R. Quisumbing:** Conceptualization, Methodology, Formal analysis, Supervision, Funding acquisition. **Kathryn Sproule:** Software, Formal analysis, Visualization. **Elena M. Martinez:** Formal analysis, Writing - review & editing. **Hazel Malapit:** Conceptualization, Methodology, Software, Formal analysis, Supervision.

## Footnotes

1 To our knowledge, the other studies using the WEAI in African countries have examined productivity outcomes and not nutrition, e.g. Diiro et al. (2018), Wouterse (2019). Kassie et al. (2020) used the abbreviated WEAI (A-WEAI) to examine the relationship between women’s empowerment and women’s dietary diversity.

2 The percentage of rural women with primary schooling in the nationally representative 2015–16 Demographic and Health Surveys is 51.9%, much lower than the percentage in our sample. This may reflect sampling for the Feed the Future Survey: in the full sample of women, not just those in our estimation sample, the percentage completing primary school is 87.1%.

3 Prior to creating the pooled weight, survey weights were normalized so each country had an average weight of one. Nepal did not have weights as it was part of an impact evaluation. Therefore, all observations from Nepal were assigned a weight of one.

4 The Bangladesh and Nepal surveys also collected information on years of schooling completed by all applicable household members. Results were robust to the specification of the women’s education variable (years of schooling, primary female years of education completed, or a dichotomous variable for literacy status). Our main specification uses a dummy for primary school completion because it is common to all countries.

5 Crosstabulations that are not reported here find that underweight girls in our sample consume fewer food groups on average (2.6 vs. 2.7 food groups) but are more likely to have the minimum diet diversity (37 vs. 36 percent) and minimum acceptable diet (21 vs. 17 percent) compared with well-nourished girls. This comparison, obtained from a slightly different estimation sample from that reported in Malapit and Quisumbing (2015), may be underpowered owing to the small sample size; these differences are not statistically significant.
