# Supplementary material for: Do tradeoffs among dimensions of women’s empowerment and nutrition outcomes exist? Evidence from six countries in Africa and Asia
Source: Food Policy. 2021 Apr;100:102001. doi: 10.1016/j.foodpol.2020.102001 (PMC8097635; doi:10.1016/j.foodpol.2020.102001)
Supplement: Supplementary data 1 [file mmc1.docx]

APPENDIX

Do tradeoffs among dimensions of women’s empowerment and nutrition outcomes exist? Evidence from six countries in Africa and Asia

Agnes R. Quisumbing^1^, Kathryn Sproule^2^, Elena M. Martinez^1,3^, Hazel Malapit^1^

^1^ International Food Policy Research Institute, Washington, DC, USA

^2^ Sproule Research Group, California, USA

^3^ Friedman School of Nutrition Science and Policy at Tufts University, Boston, MA, USA

* Corresponding author: Agnes Quisumbing

Email: [a.quisumbing@cgiar.org](mailto:a.quisumbing@cgiar.org).

**Appendix Table 1** Household characteristics, estimation sample

|  | South and Southeast Asia | | | Africa | | |
| --- | --- | --- | --- | --- | --- | --- |
| **Characteristic** | Bangladesh n=3,674 | Nepal n=2,937 | Cambodia n=1,633 | Ghana n=1,567 | Mozambique n=910 | Tanzania n=158 |
| Dual-adult household (%) | 87.30 | 37.28 | 83.50 | 75.07 | 60.94 | 64.24 |
| Primary woman completed primary school (%) | 25.49 | 10.30 | 9.04 | 7.45 | 19.22 | 79.44 |
| Age of primary women (years) | 37.62 | 43.23 | 43.50 | 45.62 | 40.18 | 39.48 |
| Household size | 4.81 | 6.28 | 5.28 | 6.38 | 5.00 | 5.69 |
| Dependency ratio | 0.91 | 1.12 | 0.66 | 1.19 | 1.25 | 1.27 |
| Woman's empowerment score | 0.67 | 0.58 | 0.98 | 0.66 | 0.80 | 0.93 |
| Intrahousehold inequality score | 0.16 | 0.25 | 0.01 | 0.20 | 0.11 | 0.03 |
| Number of: |  |  |  |  |  |  |
| Agricultural domains woman has some input in decisions or feels can make decisions | 3.09 | 3.85 | 5.67 | 3.34 | 2.42 | 5.20 |
| Agricultural domains woman has autonomy in | 2.26 | 0.47 | 3.86 | 3.25 | 2.08 | 1.58 |
| Agricultural assets woman owns | 1.49 | 1.67 | 3.51 | 0.82 | 2.38 | 3.64 |
| Agricultural assets woman can decide to buy, sell, or transfer | 2.12 | 1.92 | 3.71 | 0.92 | 2.34 | 3.75 |
| Sole or joint decisions woman has over credit | 1.23 | 0.64 | 1.75 | 0.49 | 0.16 | 1.33 |
| Income decisions woman makes | 2.53 | 2.04 | 5.72 | 1.77 | 1.78 | 3.16 |
| Groups woman is a member of | 0.31 | 2.84 | 3.36 | 2.93 | 1.11 | 1.27 |
| Contexts in which woman is comfortable speaking in public | 2.92 | 3.56 | n/a | 3.45 | 1.88 | 2.21 |
| Hours woman worked per day | 8.95 | 11.03 | 9.00 | 10.43 | n/a | 9.42 |
| Woman's satisfaction with leisure (1-10 score) | 5.21 | 3.59 | 6.34 | 6.61 | 7.26 | 6.76 |
|  |  |  |  |  |  |  |
| Household dietary diversity score | 8.49 | n/a | n/a | 3.83 | n/a | 4.33 |
|  |  |  |  |  |  |  |
| Women's dietary diversity score (15-49 years) | 4.05 | 3.83 | 3.76 | 3.83 | 3.28 | 4.43 |
| Women's BMI (15-49 years) | 20.64 | 20.22 | 21.08 | 21.34 | n/a | 22.38 |

Sources: Authors’ calculations using data from Bangladesh Integrated Household Survey (2011) for Bangladesh; baseline survey of Suaahara project for Nepal (2012); and Feed the Future surveys for Cambodia (2012), Ghana (2012), Mozambique (2012–2013), and Tanzania (2016).

*Note: Weights used. n/a indicates that the data were not available.*

Appendix Table 2. Children’s characteristics, estimation sample

|  | South and Southeast Asia | | | Africa | |
| --- | --- | --- | --- | --- | --- |
| **Characteristic** | Bangladesh n=767 | Nepal n=1,541 | Cambodia n=374 | Ghana n=733 | Mozambique n=353 |
| Female child (%) | 51.28 | 49.97 | 46.82 | 50.12 | 51.17 |
| Age of mother (years) | 27.65 | 24.52 | 29.23 | 29.00 | 28.88 |
| Height of mother (cm) | 150.29 | 151.75 | 152.62 | 158.73 | n/a |
| Mother completed primary school (%) | 40.11 | 5.14 | 40.94 | 6.27 | n/a |
| Exclusive breastfeeding (children 0-6 months, =1 if achieved, %) | n=535 | n=313 | n=74 | n=183 |  |
| Boys | 10.56 | 46.06 | 81.19 | 60.12 | n/a |
| Girls | 29.03 | 52.03 | 74.14 | 62.10 | n/a |
| All | 17.56 | 48.88 | 78.32 | 60.98 | n/a |
| Child dietary diversity score (children 6-23 months, 7 food groups) | n=767 | n=1388 | n=374 | n=504 | n=353 |
| Boys | 1.65 | 2.64 | 2.55 | 2.37 | 2.49 |
| Girls | 1.90 | 2.74 | 2.61 | 2.60 | 2.30 |
| All | 1.78 | 2.69 | 2.58 | 2.49 | 2.39 |
| Anthropometric measures (children 0-23 months) | | | | | |
| HAZ | n=767 | n=1486 | n=374 | n=665 |  |
| Boys | -1.61 | -1.46 | -1.56 | -0.99 | n/a |
| Girls | -1.51 | -1.35 | -1.20 | -0.89 | n/a |
| All | -1.56 | -1.41 | -1.39 | -0.94 | n/a |
| WHZ | n=760 | n=1479 | n=374 | n=654 |  |
| Boys | -0.44 | -0.88 | -0.54 | -0.37 | n/a |
| Girls | -0.76 | -0.86 | -0.37 | -0.56 | n/a |
| All | -0.60 | -0.87 | -0.46 | -0.47 | n/a |
| Stunted ( %) | n=767 | n=1486 | n=374 | n=665 |  |
| Boys | 39.04 | 33.51 | 38.71 | 35.03 | n/a |
| Girls | 36.38 | 31.89 | 35.44 | 28.91 | n/a |
| All | 37.67 | 32.71 | 37.18 | 32.05 | n/a |
| Wasted (%) | n=760 | n=1479 | n=374 | n=654 |  |
| Boys | 11.33 | 15.63 | 10.99 | 16.98 | n/a |
| Girls | 12.90 | 13.03 | 11.36 | 16.53 | n/a |
| All | 12.14 | 14.33 | 11.16 | 16.76 | n/a |

Sources: See notes for Table 4. No child-level outcomes were available for Tanzania. Mozambique is n/a for child-level regressions because of multicollinearity. *Notes: Weights used.*

Appendix Table 3. Household dietary diversity as a function of 10 WEAI subdomain indicators in separate regressions

|  | Coeff. | Sig. | SE | Shapley |
| --- | --- | --- | --- | --- |
| Input in productive decisions | -0.003 |  | 0.008 | 0.040 |
| Autonomy in production | 0.028 | *** | 0.010 | 0.512 |
| Ownership of assets | 0.001 |  | 0.020 | 0.669 |
| Purchase, sale or transfer of assets | 0.003 |  | 0.015 | 1.445 |
| Access to and decisions on credit | 0.010 |  | 0.015 | 0.877 |
| Control over income | 0.023 | * | 0.012 | 0.857 |
| Group membership | 0.029 |  | 0.035 | 11.098 |
| Speaking in public | -0.014 |  | 0.033 | 1.331 |
| Workload | 0.005 |  | 0.008 | 0.468 |
| Leisure | 0.026 | *** | 0.008 | 0.608 |
| Notes: n = 5,892 households in Bangladesh, Ghana and Tanzania. Household level weights are not used for the reported Shapley coefficient created by using the “rego” command in Stata. Svy estimates use household level weights (pweights). *p < 0.1, **p <0.05, ***p<0.01. | | | | |

Appendix Table 4. Women’s dietary diversity score and BMI as a function of 10 WEAI subdomain indicators in separate regressions

|  | Women’s dietary diversity | | | | Women’s BMI | | | |
| --- | --- | --- | --- | --- | --- | --- | --- | --- |
|  | Coeff. | Sig. | SE | Shapley | Coeff. | Sig. | SE | Shapley |
| Input in productive decisions | -0.035 | *** | 0.008 | 1.101 | -0.002 | *** | 0.001 | 0.548 |
| Autonomy in production | -0.059 | *** | 0.010 | 1.534 | -0.003 | ** | 0.001 | 1.528 |
| Ownership of assets | -0.036 | ** | 0.017 | 0.408 | -0.001 |  | 0.001 | 1.202 |
| Purchase, sale or transfer of assets | -0.018 |  | 0.013 | 0.220 | -0.000 |  | 0.001 | 0.857 |
| Access to and decisions on credit | -0.004 |  | 0.016 | 0.211 | 0.000 |  | 0.001 | 0.536 |
| Control over income | -0.030 | *** | 0.011 | 0.493 | -0.002 |  | 0.001 | 1.815 |
| Group membership | -0.003 |  | 0.031 | 0.313 | -0.003 |  | 0.002 | 4.890 |
| Speaking in public | 0.082 | *** | 0.027 | 4.321^[[1]](#footnote-1)^ | 0.005 | * | 0.003 | 2.539^[[2]](#footnote-2)^ |
| Workload | 0.007 |  | 0.007 | 3.658 | -0.002 | *** | 0.001 | 0.999 |
| Leisure | 0.007 |  | 0.008 | 0.818 | 0.002 | *** | 0.001 | 7.823 |
|  | | | | |  |  |  |  |

Notes: Women’s dietary diversity: n = 11,276 women 15-49 in Bangladesh, Cambodia, Nepal, Ghana, Mozambique and Tanzania. Women’s BMI: n = 9,390 women 15-49 in Bangladesh, Cambodia, Nepal, Ghana and Tanzania. Women level weights are not used for the reported Shapley coefficient created by using the “rego” command in Stata. Svy estimates use women level (pweights). *p < 0.1, **p <0.05, ***p<0.01.Appendix Table 5. Infant and young child feeding practices as a function of 10 WEAI subdomain indicators in separate regressions

|  | Exclusive breastfeeding | | | | Child dietary diversity score | | | |
| --- | --- | --- | --- | --- | --- | --- | --- | --- |
|  | Coeff. | Sig. | SE | Shapley | Coeff. | Sig. | SE | Shapley |
| **(1.a) Input in productive decisions** | 0.016 | *** | 0.006 | 3.080 | 0.004 |  | 0.018 | 0.859 |
| (1.b) Input in productive decisions x girl | -0.005 |  | 0.009 | 0.724 | -0.027 |  | 0.025 | 0.258 |
| Girl | 0.027 |  | 0.032 | 0.172 | 0.155 |  | 0.100 | 0.214 |
| *Average coeff. of empowerment: (1.a) + (1.b) * % girls* | 0.014 | *** |  |  | -0.008 |  |  |  |
| *p-value of F-test: (1.a) + (1.b) * % girls = 0* | 0.004 |  |  |  | 0.546 |  |  |  |
| **Autonomy in production** | 0.001 |  | 0.009 | 0.438 | 0.011 |  | 0.025 | 0.175 |
| Autonomy in production x girl | -0.018 |  | 0.011 | 0.266 | -0.029 |  | 0.031 | 0.088 |
| Girl | 0.046 |  | 0.033 | 0.123 | 0.120 | * | 0.072 | 0.164 |
| *Average coeff. of empowerment: (1.a) + (1.b) * % girls* | -0.008 |  |  |  | -0.003 |  |  |  |
| *p-value of F-test: (1.a) + (1.b) * % girls = 0* | 0.215 |  |  |  | 0.882 |  |  |  |
| **Ownership of assets** | 0.017 |  | 0.017 | 2.167 | 0.012 |  | 0.035 | 0.276 |
| Ownership of assets x girl | -0.007 |  | 0.020 | 0.302 | -0.048 |  | 0.045 | 0.131 |
| Girl | 0.023 |  | 0.036 | 0.121 | 0.142 |  | 0.089 | 0.193 |
| *Average coeff. of empowerment: (1.a) + (1.b) * % girls* | 0.013 |  |  |  | -0.012 |  |  |  |
| *p-value of F-test: (1.a) + (1.b) * % girls = 0* | 0.276 |  |  |  | 0.646 |  |  |  |
| **Purchase, sale or transfer of assets** | 0.002 |  | 0.012 | 0.555 | 0.009 |  | 0.027 | 0.072 |
| Purchase, sale or transfer of assets x girl | 0.015 |  | 0.016 | 0.236 | -0.069 | * | 0.037 | 0.095 |
| Girl | -0.020 |  | 0.039 | 0.096 | 0.208 | ** | 0.092 | 0.266 |
| *Average coeff. of empowerment: (1.a) + (1.b) * % girls* | 0.009 |  |  |  | -0.025 |  |  |  |
| *p-value of F-test: (1.a) + (1.b) * % girls = 0* | 0.325 |  |  |  | 0.211 |  |  |  |
| **Access to and decisions on credit** | -0.020 |  | 0.015 | 0.206 | 0.042 |  | 0.037 | 0.118 |
| Access to and decisions on credit x girl | 0.031 | * | 0.018 | 0.181 | -0.029 |  | 0.048 | 0.034 |
| Girl | -0.020 |  | 0.029 | 0.051 | 0.093 |  | 0.071 | 0.183 |
| *Average coeff. of empowerment: (1.a) + (1.b) * % girls* | -0.006 |  |  |  | 0.028 |  |  |  |
| *p-value of F-test: (1.a) + (1.b) * % girls = 0* | 0.548 |  |  |  | 0.290 |  |  |  |
| **Control over income** | 0.018 | * | 0.011 | 1.914 | 0.028 |  | 0.026 | 0.841 |
| Control over income x girl | -0.013 |  | 0.015 | 0.316 | -0.035 |  | 0.032 | 0.247 |
| Girl | 0.042 |  | 0.040 | 0.126 | 0.155 | * | 0.089 | 0.230 |
| *Average coeff. of empowerment: (1.a) + (1.b) * % girls* | 0.012 | * |  |  | 0.011 |  |  |  |
| *p-value of F-test: (1.a) + (1.b) * % girls = 0* | 0.096 |  |  |  | 0.617 |  |  |  |
| **Group membership** | -0.005 |  | 0.028 | 21.748 | 0.007 |  | 0.054 | 3.1984 |
| Group membership x girl | -0.001 |  | 0.018 | 4.284 | 0.048 |  | 0.045 | 1.0263 |
| Girl | 0.012 |  | 0.016 | 0.495 | -0.040 |  | 0.108 | 0.2086 |
| *Average coeff. of empowerment: (1.a) + (1.b) * % girls* | -0.006 |  |  |  | 0.030 |  |  |  |
| *p-value of F-test: (1.a) + (1.b) * % girls = 0* | 0.822 |  |  |  | 0.556 |  |  |  |
| **Speaking in public** | -0.015 |  | 0.028 | 2.814 | 0.082 |  | 0.075 | 1.7432 |
| Speaking in public x girl | 0.009 |  | 0.041 | 0.269 | 0.087 |  | 0.098 | 0.4724 |
| Girl | -0.014 |  | 0.124 | 0.227 | -0.202 |  | 0.327 | 0.2646 |
| *Average coeff. of empowerment: (1.a) + (1.b) * % girls* | -0.011 |  |  |  | 0.124 | ** |  |  |
| *p-value of F-test: (1.a) + (1.b) * % girls = 0* | 0.619 |  |  |  | 0.026 |  |  |  |
| **Workload** | -0.003 |  | 0.007 | 0.576 | 0.031 | ** | 0.016 | 1.321 |
| Workload x girl | -0.001 |  | 0.009 | 0.049 | -0.005 |  | 0.022 | 0.305 |
| Girl | 0.022 |  | 0.093 | 0.061 | 0.113 |  | 0.235 | 0.172 |
| *Average coeff. of empowerment: (1.a) + (1.b) * % girls* | -0.003 |  |  |  | 0.029 | ** |  |  |
| *p-value of F-test: (1.a) + (1.b) * % girls = 0* | 0.496 |  |  |  | 0.012 |  |  |  |
| **Leisure** | 0.005 |  | 0.006 | 0.325 | -0.018 |  | 0.020 | 0.132 |
| Leisure x girl | -0.013 |  | 0.009 | 0.224 | 0.011 |  | 0.028 | 0.170 |
| Girl | 0.079 |  | 0.049 | 0.184 | 0.014 |  | 0.142 | 0.134 |
| *Average coeff. of empowerment: (1.a) + (1.b) * % girls* | -0.001 |  |  |  | -0.012 |  |  |  |
| *p-value of F-test: (1.a) + (1.b) * % girls = 0* | 0.805 |  |  |  | 0.454 |  |  |  |

Notes: Exclusive breastfeeding: n = 902 children 0-6 months in Bangladesh, Cambodia, Nepal and Ghana. CDDS: n = 2,237 children 6-23 months in Bangladesh, Cambodia, Nepal, Ghana and Mozambique. Child level weights are not used for the reported Shapley coefficient created by using the “rego” command in Stata. Svy estimates use child level (pweights). *p < 0.1, **p <0.05, ***p<0.01.

Appendix Table 6. Child anthropometry as a function of 10 WEAI subdomain indicators in separate regressions

|  | HAZ | | | | WHZ | | | |
| --- | --- | --- | --- | --- | --- | --- | --- | --- |
|  | Coeff. | Sig. | SE | Shapley | Coeff. | Sig. | SE | Shapley |
| **Input in productive decisions** | 0.036 |  | 0.022 | 0.176 | -0.008 |  | 0.020 | 0.507 |
| Input in productive decisions x girl | 0.001 |  | 0.029 | 0.610 | -0.004 |  | 0.026 | 1.209 |
| Girl | 0.191 |  | 0.119 | 0.589 | -0.129 |  | 0.105 | 1.174 |
| *Average coeff. of empowerment: (1.a) + (1.b) * % girls* | 0.036 | ** |  |  | -0.009 |  |  |  |
| *p-value of F-test: (1.a) + (1.b) * % girls = 0* | 0.024 |  |  |  | 0.505 |  |  |  |
| **Autonomy in production** | -0.028 |  | 0.034 | 0.590 | -0.003 |  | 0.029 | 1.6404 |
| Autonomy in production x girl | 0.029 |  | 0.039 | 0.896 | -0.058 | * | 0.034 | 1.9876 |
| Girl | 0.139 | * | 0.083 | 0.658 | -0.040 |  | 0.073 | 1.0689 |
| *Average coeff. of empowerment: (1.a) + (1.b) * % girls* | -0.014 |  |  |  | -0.031 |  |  |  |
| *p-value of F-test: (1.a) + (1.b) * % girls = 0* | 0.585 |  |  |  | 0.157 |  |  |  |
| **Ownership of assets** | 0.012 |  | 0.041 | 0.503 | -0.011 |  | 0.037 | 0.3360 |
| Ownership of assets x girl | 0.008 |  | 0.049 | 0.207 | 0.006 |  | 0.045 | 0.4633 |
| Girl | 0.177 | * | 0.106 | 0.831 | -0.149 |  | 0.091 | 1.5821 |
| *Average coeff. of empowerment: (1.a) + (1.b) * % girls* | 0.016 |  |  |  | -0.008 |  |  |  |
| *p-value of F-test: (1.a) + (1.b) * % girls = 0* | 0.591 |  |  |  | 0.757 |  |  |  |
| **Purchase, sale or transfer of assets** | 0.049 |  | 0.033 | 0.384 | -0.017 |  | 0.028 | 0.292 |
| Purchase, sale or transfer of assets x girl | -0.002 |  | 0.042 | 0.307 | 0.004 |  | 0.037 | 1.002 |
| Girl | 0.203 | * | 0.112 | 0.876 | -0.145 |  | 0.093 | 1.297 |
| *Average coeff. of empowerment: (1.a) + (1.b) * % girls* | 0.048 | ** |  |  | -0.015 |  |  |  |
| *p-value of F-test: (1.a) + (1.b) * % girls = 0* | 0.034 |  |  |  | 0.477 |  |  |  |
| **Access to and decisions on credit** | 0.079 | * | 0.045 | 0.251 | -0.056 |  | 0.039 | 0.506 |
| Access to and decisions on credit x girl | -0.055 |  | 0.053 | 0.142 | 0.071 |  | 0.048 | 0.445 |
| Girl | 0.240 | *** | 0.083 | 1.166 | -0.200 | *** | 0.072 | 2.339 |
| *Average coeff. of empowerment: (1.a) + (1.b) * % girls* | 0.052 | * |  |  | -0.022 |  |  |  |
| *p-value of F-test: (1.a) + (1.b) * % girls = 0* | 0.075 |  |  |  | 0.405 |  |  |  |
| **Control over income** | 0.007 |  | 0.033 | 0.196 | -0.037 |  | 0.026 | 0.708 |
| Control over income x girl | 0.018 |  | 0.039 | 0.350 | 0.074 | ** | 0.032 | 0.720 |
| Girl | 0.150 |  | 0.119 | 0.669 | -0.308 | *** | 0.095 | 2.531 |
| *Average coeff. of empowerment: (1.a) + (1.b) * % girls* | 0.016 |  |  |  | -0.001 |  |  |  |
| *p-value of F-test: (1.a) + (1.b) * % girls = 0* | 0.525 |  |  |  | 0.947 |  |  |  |
| **Group membership** | 0.099 |  | 0.068 | 3.295 | -0.010 |  | 0.057 | 1.783 |
| Group membership x girl | -0.029 |  | 0.050 | 1.372 | 0.023 |  | 0.045 | 0.969 |
| Girl | 0.253 | ** | 0.123 | 0.674 | -0.190 | * | 0.114 | 1.750 |
| *Average coeff. of empowerment: (1.a) + (1.b) * % girls* | 0.085 |  |  |  | 0.001 |  |  |  |
| *p-value of F-test: (1.a) + (1.b) * % girls = 0* | 0.164 |  |  |  | 0.991 |  |  |  |
| **Speaking in public** | 0.232 | *** | 0.090 | 2.241 | -0.169 | ** | 0.077 | 2.317 |
| Speaking in public x girl | -0.175 |  | 0.113 | 0.526 | 0.176 | * | 0.099 | 1.703 |
| Girl | 0.746 | ** | 0.378 | 0.470 | -0.764 | ** | 0.340 | 1.845 |
| *Average coeff. of empowerment: (1.a) + (1.b) * % girls* | 0.148 | ** |  |  | -0.084 |  |  |  |
| *p-value of F-test: (1.a) + (1.b) * % girls = 0* | 0.020 |  |  |  | 0.136 |  |  |  |
| **Workload** | -0.001 |  | 0.022 | 0.162 | -0.000 |  | 0.016 | 0.384 |
| Workload x girl | -0.004 |  | 0.028 | 0.437 | -0.001 |  | 0.021 | 0.991 |
| Girl | 0.236 |  | 0.312 | 0.620 | -0.125 |  | 0.232 | 1.049 |
| *Average coeff. of empowerment: (1.a) + (1.b) * % girls* | -0.003 |  |  |  | -0.001 |  |  |  |
| *p-value of F-test: (1.a) + (1.b) * % girls = 0* | 0.834 |  |  |  | 0.935 |  |  |  |
| **Leisure** | 0.033 |  | 0.033 | 1.760 | 0.043 | * | 0.024 | 8.933 |
| Leisure x girl | 0.016 |  | 0.035 | 1.229 | -0.057 | ** | 0.028 | 2.326 |
| Girl | 0.114 |  | 0.162 | 0.532 | 0.133 |  | 0.143 | 1.603 |
| *Average coeff. of empowerment: (1.a) + (1.b) * % girls* | 0.041 | ** |  |  | 0.015 |  |  |  |
| *p-value of F-test: (1.a) + (1.b) * % girls = 0* | 0.038 |  |  |  | 0.399 |  |  |  |

Notes: HAZ: n = 2,483 children 0-23 months in Bangladesh, Cambodia, Nepal and Ghana. WHZ: n = 2,438 children 0-23 months in Bangladesh, Cambodia, Nepal and Ghana. Child level weights are not used for the reported Shapley coefficient created by using the “rego” command in Stata. Svy estimates use child level (pweights). *p < 0.1, **p <0.05, ***p<0.01.

Appendix Table 7. Regressions on first principal component of the 10 WEAI subdomain indicators, coefficients on empowerment index reported

| Nutrition outcomes | Coeff. | Sig. | SE | Shapley |
| --- | --- | --- | --- | --- |
| HH dietary diversity | -0.012 |  | 0.016 | 0.250 |
| Women’s dietary diversity | -0.017 |  | 0.014 | 0.087^[[3]](#footnote-3)^ |
| Women’s BMI | 0.004 | ** | 0.002 | 0.138^[[4]](#footnote-4)^ |
| Exclusive breastfeeding | -0.013 |  | 0.011 | 0.221^[[5]](#footnote-5)^ |
| EXBF * girl | -0.003 |  | 0.017 | 0.259 |
| Girl | 0.017 |  | 0.024 | 0.073 |
| *Average coeff. of empowerment: (1.a) + (1.b) * % girls* | -0.014 | * |  |  |
| *p-value of F-test: (1.a) + (1.b) * % girls = 0* | 0.098 |  |  |  |
| Children’s dietary diversity | -0.099 | *** | 0.031 | 1.300 |
| CDD * girl | 0.097 | ** | 0.043 | 0.299 |
| Girl | 0.072 |  | 0.061 | 0.213 |
| *Average coeff. of empowerment: (1.a) + (1.b) * % girls* | -0.052 | ** |  |  |
| *p-value of F-test: (1.a) + (1.b) * % girls = 0* | 0.030 |  |  |  |
| HAZ | -0.095 | ** | 0.044 | 0.856 |
| HAZ * girl | 0.054 |  | 0.056 | 0.126 |
| Girl | 0.150 | ** | 0.072 | 0.717 |
| *Average coeff. of empowerment: (1.a) + (1.b) * % girls* | -0.068 | ** |  |  |
| *p-value of F-test: (1.a) + (1.b) * % girls = 0* | 0.026 |  |  |  |
| WHZ | -0.018 |  | 0.038 | 0.275 |
| WHZ * girl | -0.009 |  | 0.047 | 0.126 |
| Girl | -0.174 | *** | 0.062 | 2.843 |
| *Average coeff. of empowerment: (1.a) + (1.b) * % girls* | -0.023 |  |  |  |
| *p-value of F-test: (1.a) + (1.b) * % girls = 0* | 0.372 |  |  |  |
| Notes: See individual tables for household, women and child level sample sizes and included countries. Weights are not used for the reported Shapley coefficient created by using the “rego” command in Stata. Some country dummies were not included in the Shapley estimates because of multicollinearity. Svy estimates are weighted (pweights). *p < 0.1, **p <0.05, ***p<0.01. | | | | |

Appendix Table 8. Women’s empowerment, intrahousehold inequality, and household and women’s dietary and nutrition outcomes, selected coefficients

|  | Household outcomes | Women’s outcomes | |
| --- | --- | --- | --- |
|  | HH dietary diversity score | Women’s dietary diversity score | Women’s BMI |
| **Bangladesh** |  |  |  |
| Model 1: Empowerment score | 0.252** | 0.284* | -0.038** |
|  | (0.115) | (0.150) | (0.016) |
| Model 2: Intrahousehold inequality score | -0.398*** | -0.317* | 0.029 |
|  | (0.127) | (0.168) | (0.019) |
| N | 4,283 | 4,494 | 4,102 |
| **Nepal** |  |  |  |
| Model 1: Empowerment score |  | 0.098 | 0.062*** |
|  |  | (0.128) | (0.015) |
| Model 2: Intrahousehold inequality score |  | 0.099 | -0.057*** |
|  |  | (0.161) | (0.019) |
| N |  | 2,031 | 1,887 |
| **Cambodia** |  |  |  |
| **Model 1: Empowerment score** |  | 0.164 | 0.018 |
|  |  | (0.553) | (0.037) |
| **Model 2: Intrahousehold inequality score** |  | -1.236 | -0.049 |
|  |  | (1.436) | (0.089) |
| N |  | 2,800 | 2,285 |
| **Ghana** |  |  |  |
| Model 1: Empowerment score | -0.277 | 0.251 | 0.003 |
|  | (0.252) | (0.247) | (0.018) |
| Model 2: Intrahousehold inequality score | 0.425 | -0.147 | 0.015 |
|  | (0.301) | (0.236) | (0.021) |
| N | 1,997 | 2,123 |  |
| **Mozambique** |  |  |  |
| Model 1: Empowerment score |  | 0.382 |  |
|  |  | (0.265) |  |
| Model 2: Intrahousehold inequality score |  | -0.596 |  |
|  |  | (0.457) |  |
| N |  | 1,210 |  |
| **Tanzania** |  |  |  |
| Model 1: Empowerment score | 0.781 | 0.056 | 0.106 |
|  | (0.596) | (0.893) | (0.081) |
| Model 2: Intrahousehold inequality score | -2.540 | -4.595* | -0.145 |
|  | (1.708) | (2.412) | (0.186) |
| N | 249 | 238 | 179 |

Notes: * p < 0.10, ** p < 0.05, *** p < 0.01. Standard errors in parentheses

Appendix Table 9. Women’s empowerment, intrahousehold inequality, child anthropometric outcomes, and infant and young child feeding practices

| **Empowerment indicators** | Child anthropometric outcomes | | Infant and young child feeding practices | |
| --- | --- | --- | --- | --- |
|  | HAZ | WHZ | EBF | CDDS |
| **Bangladesh** |  |  |  |  |
| **Model 1: Empowerment score** | 0.458 | 0.189 | 0.042 | 0.037 |
|  | (0.425) | (0.431) | (0.040) | (0.291) |
| *Empowerment score x Girl* | -0.720 | 0.003 | -0.034 | 0.119 |
|  | (0.631) | (0.544) | (0.055) | (0.431) |
| *Girl* | 0.661 | -0.294 | 0.050 | -0.007 |
|  | (0.407) | (0.369) | (0.040) | (0.268) |
| **Model 2: Intrahousehold inequality score** | -0.609 | -0.541 | -0.069* | 0.038 |
|  | (0.505) | (0.478) | (0.039) | (0.299) |
| *Intrahousehold inequality score x Girl* | 1.161* | 0.343 | 0.048 | -0.309 |
|  | (0.619) | (0.608) | (0.042) | (0.453) |
| *Girl* | 0.018 | -0.366** | 0.010 | 0.139 |
|  | (0.190) | (0.176) | (0.018) | (0.152) |
| N | 738 | 731 | 525 | 726 |
| **Nepal** |  |  |  |  |
| **Model 1: Empowerment score** | 0.610** | -0.150 | 0.096 | 0.238 |
|  | (0.246) | (0.288) | (0.185) | (0.253) |
| *Empowerment score x Girl* | -0.204 | 0.154 | -0.074 | -0.064 |
|  | (0.324) | (0.366) | (0.259) | (0.330) |
| *Girl* | 0.300 | -0.092 | 0.048 | 0.093 |
|  | (0.193) | (0.209) | (0.162) | (0.193) |
| **Model 2: Intrahousehold inequality score** | -0.093 | 0.226 | -0.141 | 0.310 |
|  | (0.315) | (0.339) | (0.242) | (0.302) |
| *Intrahousehold inequality score x Girl* | 0.036 | -0.250 | 0.049 | -0.413 |
|  | (0.417) | (0.400) | (0.308) | (0.392) |
| *Girl* | 0.245* | 0.035 | -0.031 | 0.128 |
|  | (0.145) | (0.138) | (0.110) | (0.145) |
| N | 1,030 | 1,025 | 229 | 956 |
| **Cambodia** |  |  |  |  |
| **Model 1: Empowerment score** | -0.338 | 1.535* | 1.578*** | -0.099 |
|  | (0.893) | (0.895) | (0.509) | (0.794) |
| *Empowerment score x Girl* | 2.395 | -0.963 | -1.038 | 0.151 |
|  | (1.442) | (1.399) | (0.678) | (1.087) |
| *Girl* | -1.974 | 0.994 | 0.984 | -0.103 |
|  | (1.384) | (1.351) | (0.666) | (1.007) |
| **Model 2: Intrahousehold inequality score** | 1.748 | -1.677 | -1.622* | -0.970 |
|  | (1.831) | (2.049) | (0.874) | (1.410) |
| *Intrahousehold inequality score x Girl* | -2.523 | 1.911 | -2.411 | 2.739 |
|  | (3.157) | (3.009) | (2.216) | (2.330) |
| *Girl* | 0.344** | 0.170 | -0.112 | 0.016 |
|  | (0.171) | (0.156) | (0.079) | (0.178) |
| N | 353 | 353 | 78 | 365 |
| **Ghana** |  |  |  |  |
| **Model 1: Empowerment score** | 0.868 | 0.063 | 0.060 | 1.318*** |
|  | (0.551) | (0.432) | (0.254) | (0.480) |
| *Empowerment score x Girl* | -2.310** | 0.875 | -0.047 | -2.606*** |
|  | (1.022) | (0.768) | (0.292) | (0.687) |
| *Girl* | 1.710** | -0.748 | 0.072 | 1.858*** |
|  | (0.675) | (0.498) | (0.219) | (0.494) |
| **Model 2: Intrahousehold inequality score** | -1.046 | 0.210 | -0.169 | -1.401** |
|  | (0.636) | (0.507) | (0.305) | (0.559) |
| *Intrahousehold inequality score x Girl* | 2.275** | -1.251 | 0.026 | 2.754*** |
|  | (0.949) | (0.925) | (0.384) | (0.788) |
| *Girl* | -0.339 | 0.102 | 0.021 | -0.415 |
|  | (0.323) | (0.341) | (0.111) | (0.260) |
| N | 638 | 628 | 180 | 490 |
| **Mozambique** |  |  |  |  |
| **Model 1: Empowerment score** | n.a. | n.a. | n.a. | 0.277 |
|  |  |  |  | (0.442) |
| *Empowerment score x Girl* | n.a. | n.a. | n.a. | 0.506 |
|  |  |  |  | (0.603) |
| *Girl* | n.a. | n.a. | n.a. | -0.452 |
|  |  |  |  | (0.475) |
| **Model 2: Intrahousehold inequality score** | n.a. | n.a. | n.a. | -0.521 |
|  |  |  |  | (0.678) |
| *Intrahousehold inequality score x Girl* | n.a. | n.a. | n.a. | 0.217 |
|  |  |  |  | (0.913) |
| *Girl* | n.a. | n.a. | n.a. | -0.044 |
|  |  |  |  | (0.205) |
| N |  |  |  | 310 |

Notes: * p < 0.10, ** p < 0.05, *** p < 0.01. Standard errors in parentheses

1. Cambodia and Mozambique country dummies dropped due to multicollinearity. Cambodia does not collect speaking in public. [↑](#footnote-ref-1)
2. Cambodia and Mozambique country dummies dropped due to multicollinearity. Cambodia does not collect speaking in public. Mozambique does not collect BMI. [↑](#footnote-ref-2)
3. Cambodia and Mozambique country dummies dropped due to multicollinearity. [↑](#footnote-ref-3)
4. Cambodia country dummy dropped due to multicollinearity. [↑](#footnote-ref-4)
5. Cambodia country dummy dropped due to multicollinearity. [↑](#footnote-ref-5)
